# Associations between sex hormones, receptors, binding proteins and inflammatory bowel disease: a Mendelian randomization study

**DOI:** 10.3389/fendo.2024.1272746

**Published:** 2024-04-10

**Authors:** Fei Zou, Yaxian Hu, Mengmeng Xu, Su Wang, Zengrong Wu, Feihong Deng

**Affiliations:** ^1^ Department of Gastroenterology, The Second Xiangya Hospital, Central South University, Changsha, Hunan, China; ^2^ Research Center of Digestive Disease, Central South University, Changsha, Hunan, China; ^3^ Department of Neurology, The Second Xiangya Hospital, Central South University, Changsha, Hunan, China

**Keywords:** inflammatory bowel disease, sex hormone, estradiol, progesterone, testosterone, Mendelian randomization

## Abstract

**Background:**

Gender differences existed in inflammatory bowel disease (IBD), including Crohn’s disease (CD) and ulcerative colitis (UC). Observational studies have revealed associations between sex hormones and IBD, such as estrogen and testosterone. However, the exact relationship between these sex hormones and IBD is unclear.

**Method:**

Based on the genome-wide association studies data of eight sex hormones, two sex hormone receptors, sex hormone-binding globulin (SHBG), total IBD and its two subtypes, we performed a two-sample Mendelian randomization (MR) study to analyze their mutual relationship. For estradiol (E2), progesterone (PROG), bioavailable testosterone (BAT), total testosterone (TT) and SHBG, sex-stratified MR analyses were also performed. Inverse variance weighted method, MR-Egger regression and Weighted median method were used for causal analyses. Sensitivity analyses were conducted to test the stability of causal relationships. Besides, a reverse MR analysis was performed to estimate the reverse causation.

**Results:**

E2 (*P*=0.028) and TT (*P*=0.034) had protective effects on CD. Sex-stratified analyses revealed protective roles of E2 in males on total IBD (*P*=0.038) and CD (*P*=0.020). TT in females had protective effects on total IBD (*P*=0.025) and CD (*P*=0.029), and BAT in females decreased the risk of developing CD (*P*=0.047) and UC (*P*=0.036). Moreover, SHBG in males was also associated with a decreased risk of CD (*P*=0.021). The reversed MR analysis showed that CD was negatively correlated with estrogen receptor (*P*=0.046). UC was negatively correlated with PROG in females (*P*=0.015) and positively correlated with SHBG levels in males (*P*=0.046).

**Conclusion:**

Findings of this study revealed the mutual causal associations between sex hormones and the risk of developing IBD.

## 1 Introduction

Inflammatory bowel disease (IBD) is a group of chronic non-specific inflammatory disorders of the gastrointestinal tract, including ulcerative colitis (UC) and Crohn’s disease (CD), the etiology of which remains unclear. The pathogenesis of IBD is believed to be multifactorial, involving genetic factors, environmental factors, dysbiosis of the intestinal microbiota, and immune dysregulation (1). Sex hormones with their receptors and binding proteins have garnered significant attention due to their widespread distribution and diverse effects throughout the body, especially their immunoregulatory effects which play key roles in autoimmune diseases, including systemic lupus erythematosus (2), rheumatoid arthritis (3), etc. Estrogen, progesterone (PROG) and androgens, as three main sex hormones, modulate immunocytes proliferation, differentiation and activation (4). While estrogen in general has immunostimulatory roles, PROG and androgens are immunosuppressive and counteract the pathways affected by estrogen (5). Therefore, elucidating the relationship between sex hormones and IBD is helpful for further unraveling the pathogenesis of IBD.

Gender disparities in IBD incidence and the impact of pregnancy on IBD suggest that sex hormones may influence the susceptibility of IBD. Epidemiological studies have revealed a gender difference in CD, whereas the gender difference in the epidemiology of UC is less pronounced (6). Early-onset CD (<16 years) is more common in males, with an increased risk of CD in males aged 10-14 compared to females (7, 8). However, females with an age of 25-29 years and older than 35 years have a higher risk of CD compared to males (8). Males over age 45 have a higher risk of UC compared to females (8). Furthermore, the effects of sex hormones on IBD during pregnancy may be linked to the functions of estrogen and PROG which are increased during pregnancy. Several studies revealed a more severe disease course during pregnancy in female patients with UC, and women previously diagnosed UC had an increased risk of clinical flares during pregnancy (9, 10). By contrast, pregnancy plays a more positive role in CD patients. More women with CD reported improvement in symptoms during pregnancy than women with UC (4). These studies indicate that the exposure to varying levels of different sex hormones at different stages of life may cause varied effects to the onset and progression of IBD.

Several cross-sectional studies have demonstrated the changes in the levels of sex hormones among patients with IBD. For UC, in male patients in remission, there are no significant differences in serum levels of follicle-stimulating hormone (FSH), luteinizing hormone (LH), and total testosterone (TT) compared to healthy men (11). However, studies have indicated a negative correlation between testosterone levels and the extent of rectal bleeding in male UC patients (12). For CD, in male patients, serum levels of testosterone, estradiol (E2) and sex hormone binding globulin (SHBG) are lower than those in healthy individuals (13). Moreover, for CD patients in remission, there is a decline in male FSH and LH levels, accompanied by an increase in TT levels (14). In female CD patients, serum levels of anti-Müllerian hormone (AMH) are lower than those in healthy individuals with a negative correlation with disease activity (15, 16), suggesting diminished ovarian reserve in active CD patients. Moreover, the expressions of estrogen receptor (ER) in the intestinal mucosa and peripheral blood T lymphocytes are reduced in IBD patients (17, 18). These findings suggest a significant association between sex hormones and IBD.

The modulatory mechanisms of sex hormones in the pathogenesis of IBD have been investigated. Sex hormones play a pivotal role in modulating the abundance of gut microbiota, protecting intestinal epithelial barrier function, and regulating mucosal immune response (4). Several clinical studies have observed the effects of exogenous supplementation of sex hormones on the development and progression of IBD. Women of childbearing age who use oral contraceptives have a higher risk of both CD and UC compared to those who do not take them (9). However, Kane et al. found that postmenopausal women with IBD who received hormone replacement therapy experienced significantly reduced disease activity (19), indicating potential differences in the effects of exogenous estrogen and PROG on IBD among different age groups and IBD subtypes. Male CD patients treated with exogenous testosterone show significantly reduced disease activity compared to the control group (20), suggesting a protective role of androgen on male CD. In conclusion, based on the current clinical studies, the impact of sex hormones on IBD is uncertain and complicated. The effects of sex hormones may vary across different genders, age groups and IBD subtypes.

Mendelian randomization (MR) is a bioinformatics analytical method that utilizes genome-wide association studies (GWAS) data to investigate the causal associations. MR analysis uses single nucleotide polymorphisms (SNP) as instrumental variables (IVs) to replace exposure factors, which effectively balances the influences of confounding factors. Generally, MR studies involve large sample sizes and it can avoid the reverse causation often seen in observational epidemiological studies, thus yielding results with higher credibility. Recently, multiple MR studies related to IBD have been reported, confirming various risk factors for IBD such as depression (21), diet (22), smoking and vitamin D deficiency (23). Furthermore, MR studies have also demonstrated associations between sex hormones and SHBG with hypertension, diabetes and coronary heart disease with gender differences (24). However, to date, the MR studies exploring the causal relationship between sex hormones and IBD is lacked. The present study performs MR analysis to explore the genetically predicted impact of sex hormones, sex hormone receptors and SHBG on the incidence of IBD. Additionally, a sex-stratified analysis is conducted to reveal potential differences in these causal associations across different genders. Moreover, a reverse MR analysis is performed to investigate the influence of IBD on sex hormone levels in patients.

## 2 Methods

### 2.1 Data sources

GWAS data of FSH, LH, ER and prolactin receptor (PRLR) were obtained from a genomic atlas study of the human plasma proteome including 3,301 European participants (25). Summary statistics for E2 that included 67,623 participants (93.7% European) were obtained from Pan-UK Biobank. Sex-stratified GWAS data of E2 were obtained from a large-scale study containing 147,690 European males and 163,985 European females (26). GWAS data on PROG has been contributed by LIFE-Adult and LIFE-Heart investigators (27). Non-stratified GWAS data on PROG included 2,070 Europeans, while sex-stratified GWAS data on PROG included 1,358 European males and 1,261 European females. GWAS summary statistics for prolactin (PRL) were extracted from a study that included 30,931 Europeans (28). GWAS data of bioavailable testosterone (BAT), TT and SHBG was obtained from a large-scale study containing UK Biobank study participants (29). Specifically, the sample size of BAT GWAS data was 382,988 (non-stratified), 178,782 (male) and 188,507 (female). The sample size of TT GWAS data was 425,097 (non-stratified), 194,453 (male) and 230,454 (female). The sample size of SHBG GWAS data was 370,125 (non-stratified), 180,726 (male) and 189,473 (female). Summary statistics for AMH were extracted from a GWAS meta-analysis, including 7,049 female participants of European ancestry (30). SNPs-outcome associations were retrieved from the biggest GWAS reported for IBD to date (31). IBD GWAS included 12,882 cases and 21,770 controls. CD GWAS included 5,956 cases and 14,927 controls. UC GWAS included 6,968 cases and 20,464 controls. All participants are Europeans. Diagnosis of IBD was based on accepted radiologic, endoscopic, and histopathologic evaluation. The details of dataset were listed in **Supplementary Table S1**.

### 2.2 IVs selection

**Figure 1:**
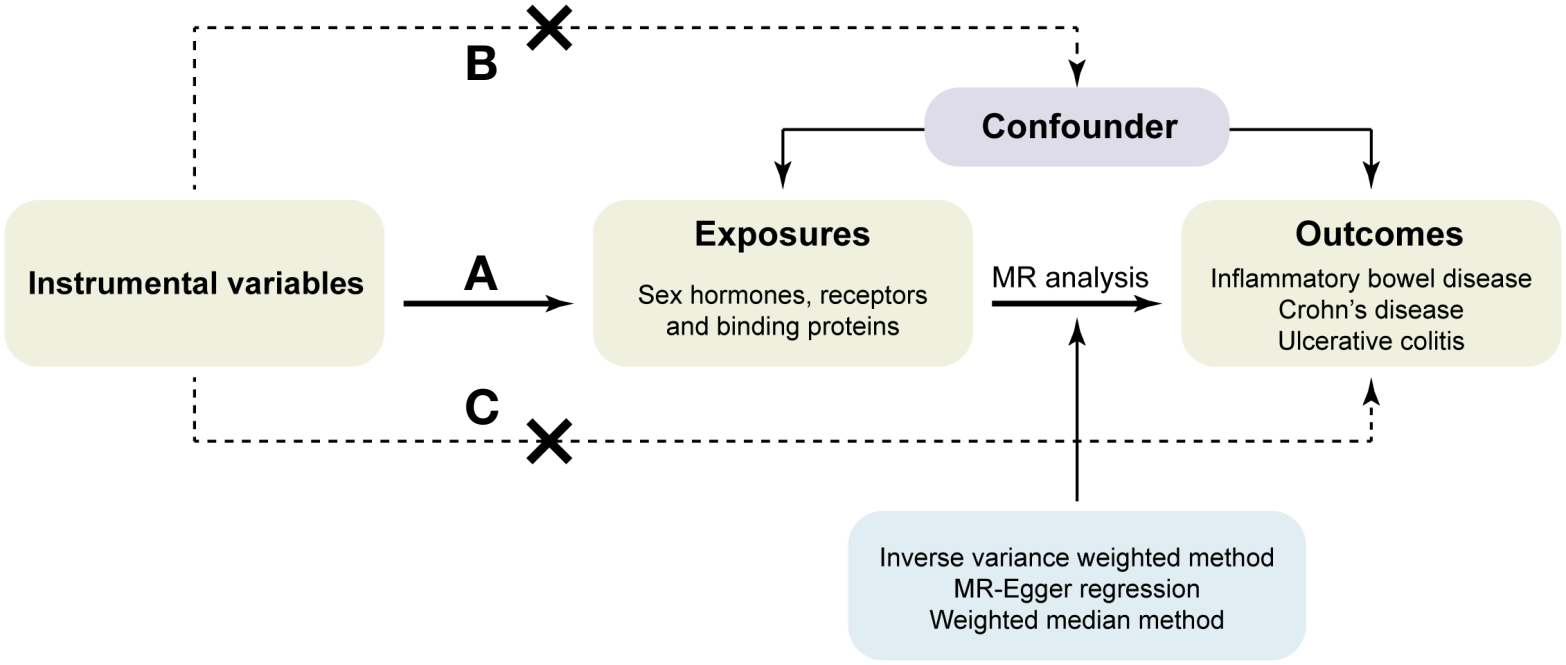
Flowchart of the Mendelian randomization study design.

According to the principles of MR, the IVs used for analysis must meet the following three assumptions (32): (a) IVs are strongly associated with the exposure. (b) IVs are not associated with confounding factors. (c) IVs affect the risk of outcomes directly through exposure, not through other pathways (**Figure 1**).

As the mediators between exposure factors and outcomes to study the causal relationship in the MR analysis, SNPs were selected as IVs only if they meet the following criteria: (a) SNPs were intensely related with exposures at the genome-wide significance level (*P*<5×10^-8^). However, if there were less than two available SNPs after clumping to eliminate the linkage disequilibrium, the threshold for *P* value was enlarged to 5×10^-6^. (b) No linkage disequilibrium was among the included IVs as the linkage disequilibrium correlation coefficient was set to r^2^<0.001 and clumping window>10,000kb. (c) The F-statistic was used to estimate the association between IVs and exposures in order to avoid the weak instrument bias. Only when the F-statistic was greater than 10 can the IV be considered strong associated with the exposure and used for MR analysis. The F-statistic was calculated using the following formula: $F=\beta_{exposure}^{2}/SE_{exposure}^{2}$ (33). Besides, before MR analysis, we conducted harmonization to exclude palindromic SNPs and ensure that the alleles of each SNP were consistent between the exposure and outcome.

### 2.3 Mendelian randomization analysis

The main MR analysis used in this study was the inverse variance weighted (IVW) method (34), which is the most effective method in the presence of valid IVs. Because the random-effects and fixed-effect results will be identical if there is no excess heterogeneity and the fixed-effect estimate is inappropriate if there is excess heterogeneity, we applied random-effects IVW method for MR analysis (35), thus heterogeneity can also be taken into account when analyzing causality (36). Besides, two additional methods were employed: the MR-Egger regression (37) and the weighted median method (38). The MR-Egger regression adjusts for horizontal pleiotropy by pooling a single SNP-specific Wald ratio through adaptive Egger regression, and the weighted median method uses the weighted median of Wald ratios, provided that at least 50% of the variants meet a valid IV for the exclusion restrictions (36).

Based on the MR models mentioned above and the previous study (39), we considered a relatively robust causal relationship meeting the following criteria: (a) IVW analysis presented a significant correlation (*P*<0.05); (b) MR analyses presented consistent directions of causal estimates among all three methods. The two-sample MR analysis was performed using the “TwoSampleMR” package (version 0.5.6) in R software (version 4.2.3).

### 2.4 Sensitivity analysis

Sensitivity analysis was performed as described previously (40). Cochran’s Q test was used to detect the presence of heterogeneity. The analytical result with a *P* value more than 0.05 was considered as no significant heterogeneity. Presence of heterogeneity is acceptable in MR analysis and it do not invalidate the MR estimates because the random-effect IVW method might balance the pooled heterogeneity (39). MR pleiotropy residual sum and outlier (MR-PRESSO) test (41) was used to detect the presence of outlying IVs. The residual sum of squares is a measure of heterogeneity, and is equal to Cochran’s Q statistic (35). When there were outlying IVs, MR-PRESSO outlier-corrected test was used to obtain corrected causal effects by excluding outliers and MR-PRESSO distortion test was used to test the distortion in the causal estimates before and after outlier removal. The MR-PRESSO test will be valuable when there is a small number of genetic variants with heterogeneous ratio estimates, as they will be removed from the analysis, and so will not influence the overall estimate (35). MR-Egger intercept (37) test was used to detect the presence of horizontal pleiotropy. The analytical result with a *P* value more than 0.05 suggested that the intercept term was centered around the origin and there was no evidence of horizontal pleiotropy. MR-PRESSO test and MR-Egger intercept test are unavailable when there are few IVs. However, the causal associations are less likely to be affected by pleiotropy and heterogeneity in this case (42).

### 2.5 Ethical approval

The GWAS data used in this study were all obtained from publicly available databases and were approved by the ethics committee. Thus, this two-sample MR analysis do not need additional ethical approval.

## 3 Results

### 3.1 Selection of instrumental variables

SNPs significantly associated with sex hormone-related risk factors or IBD were selected as IVs based on the aforementioned principles. The numbers of IVs for each trait were shown in **Supplementary Table S1**. The F-statistic of each IV was above the threshold of 10, indicating the absence of weak instrument bias.

### 3.2 The causal effects of sex hormones, receptors and binding proteins on IBD

**Figure 2:**
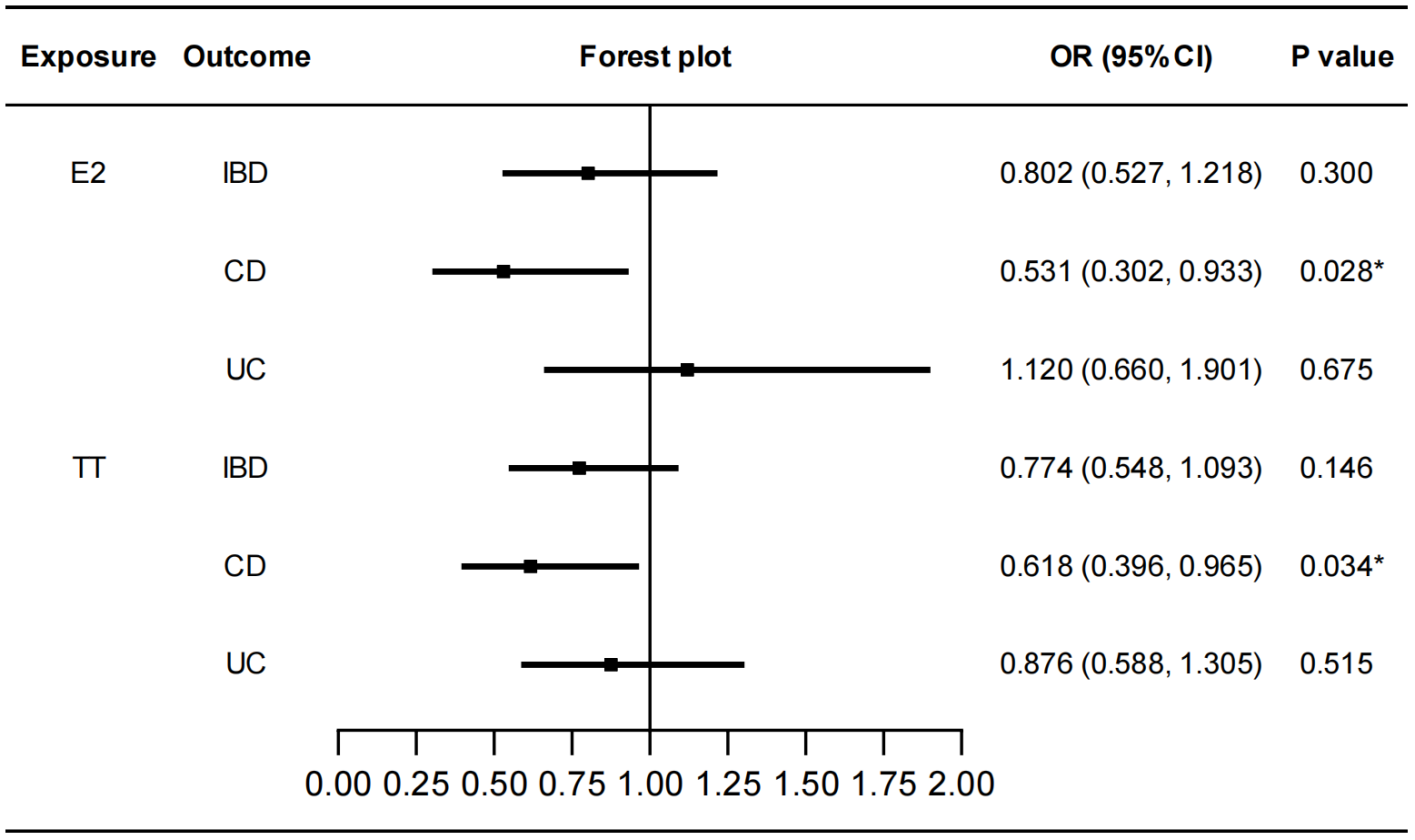
Causal effects of E2 and TT on IBD/CD/UC. Forest plots were used to show the MR estimate and 95% CI values using the inverse variance weighted method. *P<0.05. E2, estradiol; TT, total testosterone; IBD, inflammatory bowel disease; CD, Crohn’s disease; UC, ulcerative colitis; OR, odds ratio; CI, confidence interval.

We performed MR analysis to identify the genetically predicted effects of seven sex hormones (FSH, LH, E2, PROG, PRL, BAT, and TT), two sex hormone receptors (ER and PRLR) and SHBG on IBD and its two subtypes (CD and UC). Protective effects of E2 on CD were detected (IVW: OR=0.531, 95%CI: 0.302, 0.933, *P*=0.028) with no heterogeneity or pleiotropy (**Figure 2**; **Supplementary Table S2**). TT also had a protective effect on CD (IVW: OR=0.618, 95%CI: 0.396, 0.965, *P*=0.034) (**Figure 2**). The Cochran’s Q test showed significant heterogeneity (*P*<0.001). However, the causal effect still existed after outliers were removed using MR-PRESSO test (Outlier-corrected: *P*=0.017, Distortion test: *P*=0.928) and the MR-Egger intercept test showed no pleiotropy (**Supplementary Table S2**), suggesting the robustness of the correlation. The results of MR-Egger regression and weighted median remained directionally consistent with that of IVW method. Besides, no causal associations were found between FSH, LH, PROG, PRL, BAT and IBD (**Supplementary Figure S1**). Details of the causal analysis using all three methods were shown in **Supplementary Table S3**. The scatter plots of causal relationships were shown in **Supplementary Figure S2**.

No genetically predicted effects of ER, PRLR and SHBG on IBD were found (**Supplementary Figure S3**). Details of the causal analysis using all three methods were shown in **Supplementary Table S4**. The scatter plots of causal relationships were shown in **Supplementary Figure S4**. The Cochran’s Q test showed significant heterogeneity for ER-IBD, ER-CD, SHBG-IBD, SHBG-CD and SHBG-UC correlations (**Supplementary Table S5**). However, after removing outliers, no significant causal relationships were revealed by the MR-PRESSO test. No pleiotropy was detected by the MR-Egger intercept test (**Supplementary Table S5**).

### 3.3 Sex-stratified causal effects of E2, PROG, BAT, TT, SHBG and AMH on IBD

**Figure 3:**
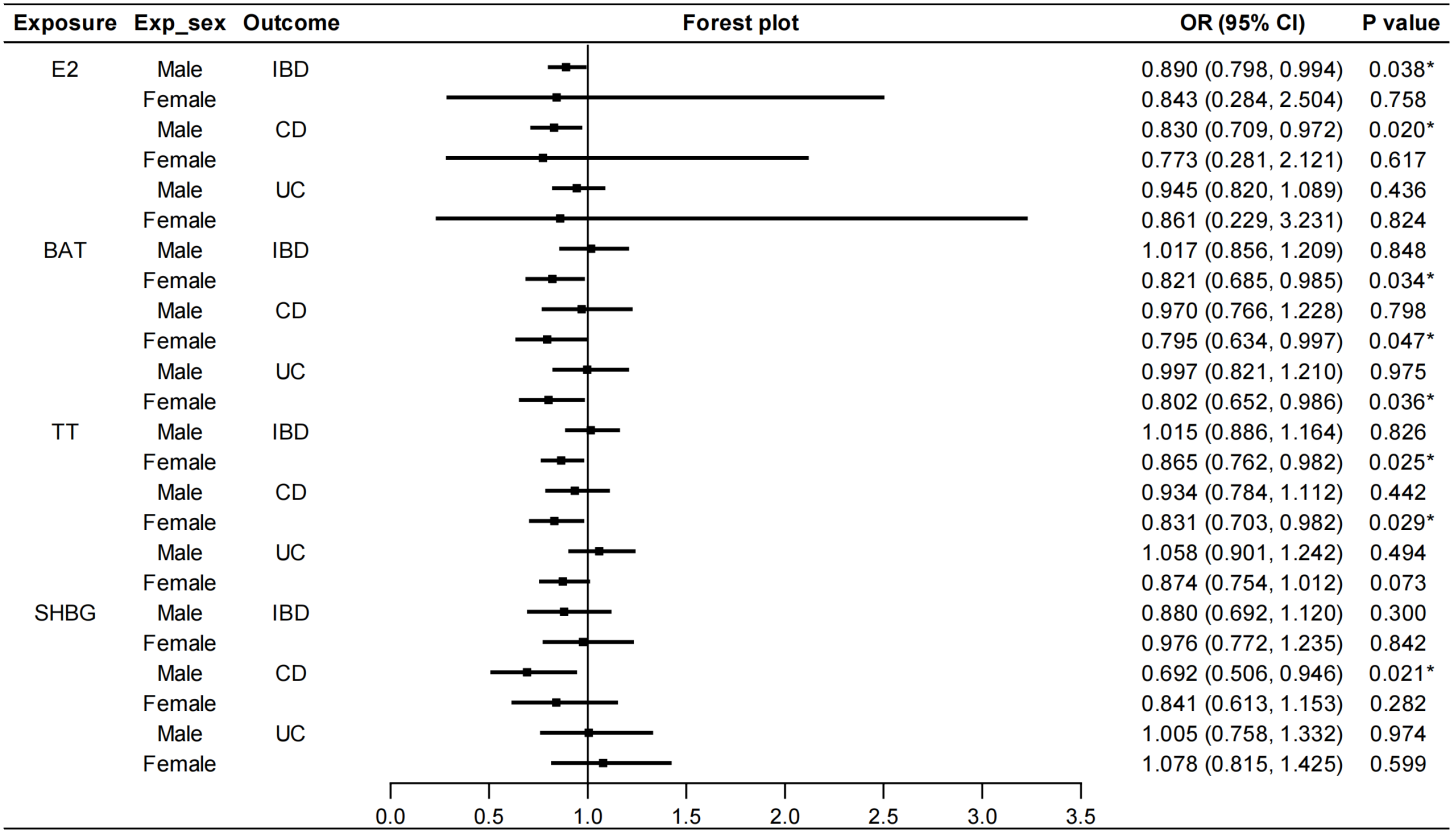
Sex-stratified causal effects of E2, BAT, TT and SHBG on IBD/CD/UC. Forest plots were used to show the MR estimate and 95% CI values using the inverse variance weighted method. *P<0.05. E2, estradiol; BAT, bioavailable testosterone; TT, total testosterone; SHBG, sex hormone binding globulin; IBD, inflammatory bowel disease; CD, Crohn’s disease; UC, ulcerative colitis; OR, odds ratio; CI, confidence interval.

We stratified the MR analyses by sex for E2, BAT, TT and SHBG in order to exclude the bias caused by sex. As shown in **Figure 3**, E2 in males had a protective effect on IBD (IVW: OR=0.890, 95%CI: 0.798, 0.994, *P*=0.038). Specifically, E2 in males decreased the risk of CD (IVW: OR=0.830, 95%CI: 0.709, 0.972, *P*=0.020) but not UC. This protective role was not affected by heterogeneity or pleiotropy (**Supplementary Table S6**). Similarly, TT in females had protective effects on IBD (IVW: OR=0.865, 95%CI: 0.762, 0.982, *P*=0.025) and CD (IVW: OR=0.831, 95%CI: 0.703, 0.982, *P*=0.029). Heterogeneity was found but outliers did not affect the stability of causal relationships between TT in females and IBD (Outlier-corrected: *P*=0.041, Distortion test: *P*=0.663) and CD (Outlier-corrected: *P*=0.002, Distortion test: *P*=0.488). BAT in females decreased the risk of both CD (IVW: OR=0.795, 95%CI: 0.634, 0.997, *P*=0.047) and UC (IVW: OR=0.802, 95%CI: 0.652, 0.986, *P*=0.036). Although heterogeneity was revealed by Cochran’s Q test, no significant outliers were detected by the MR-PRESSO test. Moreover, SHBG in males was also associated with a decreased risk of CD (IVW: OR=0.692, 95%CI: 0.506, 0.946, *P*=0.021). Also, the outliers did not affect this correlation (Outlier-corrected: *P*=0.016, Distortion test: *P*=0.980) although heterogeneity was detected. No pleiotropy were found in the above MR analyses and the results of IVW, MR-Egger regression and weighted median were in the consistent direction. Besides, PROG in either males or females was not causally associated with IBD/CD/UC. AMH in females had no causal effects on IBD/CD/UC (**Supplementary Figure S5**). Because there was no accessible GWAS data for AMH in males, the causal effect of AMH in males on IBD was not analyzed. Details of the causal analysis using all three methods were shown in **Supplementary Tables S7–S12**. The scatter plots of causal relationships were shown in **Supplementary Figures S6–S11**.

### 3.4 The causal effects of IBD on sex hormones, receptors and binding proteins

**Figure 4:**
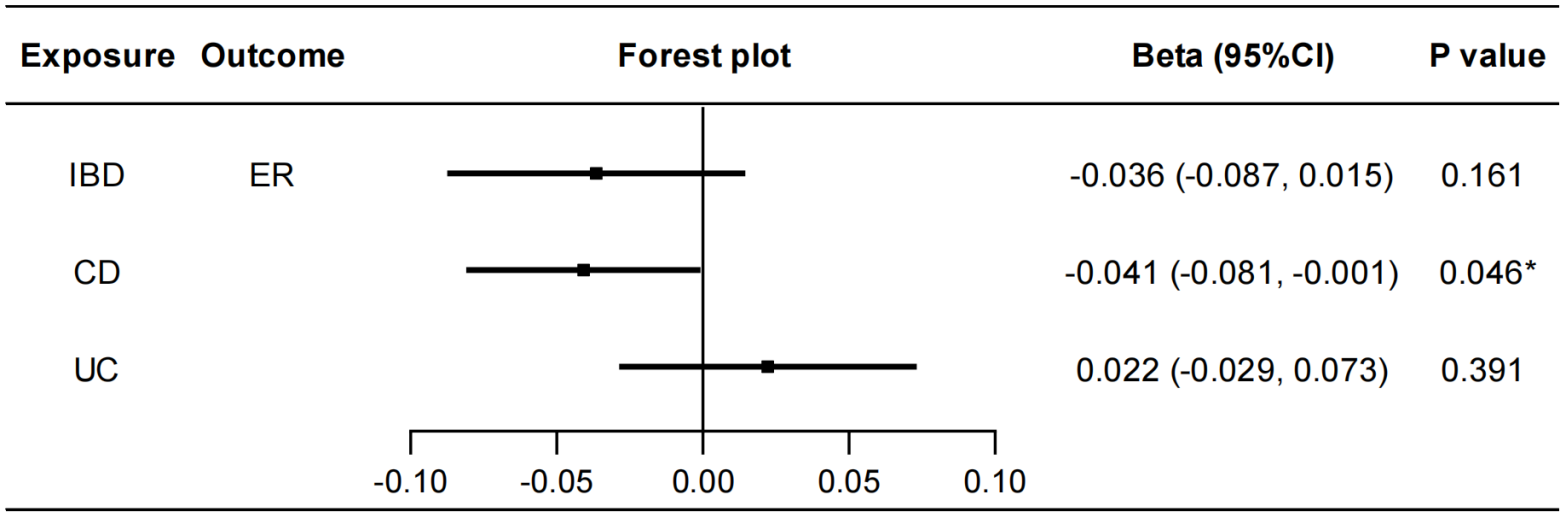
Causal effects of IBD/CD/UC on ER. Forest plots were used to show the MR estimate and 95% CI values using the inverse variance weighted method. *P<0.05. IBD, inflammatory bowel disease; CD, Crohn’s disease; UC, ulcerative colitis; ER, estrogen receptor; CI, confidence interval.

To explore whether IBD has any causal effect on sex hormones, receptors and binding proteins, a reverse MR analysis was performed. No genetically predicted effects of IBD as well as CD and UC on the seven sex hormones (FSH, LH, E2, PROG, PRL, BAT, and TT) were found (**Supplementary Figure S12**). As shown in **Figure 4**, the IVW method showed that CD was negatively correlated with ER (beta=-0.041, 95%CI: -0.081, -0.001, *P*=0.046), and the results of other two methods, MR-Egger regression and weighted median, were in the same direction with the IVW result. The Cochran’s Q test, MR-PRESSO test and MR-Egger intercept test showed this robust correlation was not affected by heterogeneity or pleiotropy. Besides, IBD had no causal effects on PRLR and SHBG (**Supplementary Figure S13**). Details of the causal analysis and sensitive analysis were shown in **Supplementary Tables S13–S16**. The scatter plots of causal relationships were shown in **Supplementary Figures S14, S15**.

### 3.5 Sex-stratified causal effects of IBD on E2, PROG, BAT, TT, SHBG and AMH

**Figure 5:**
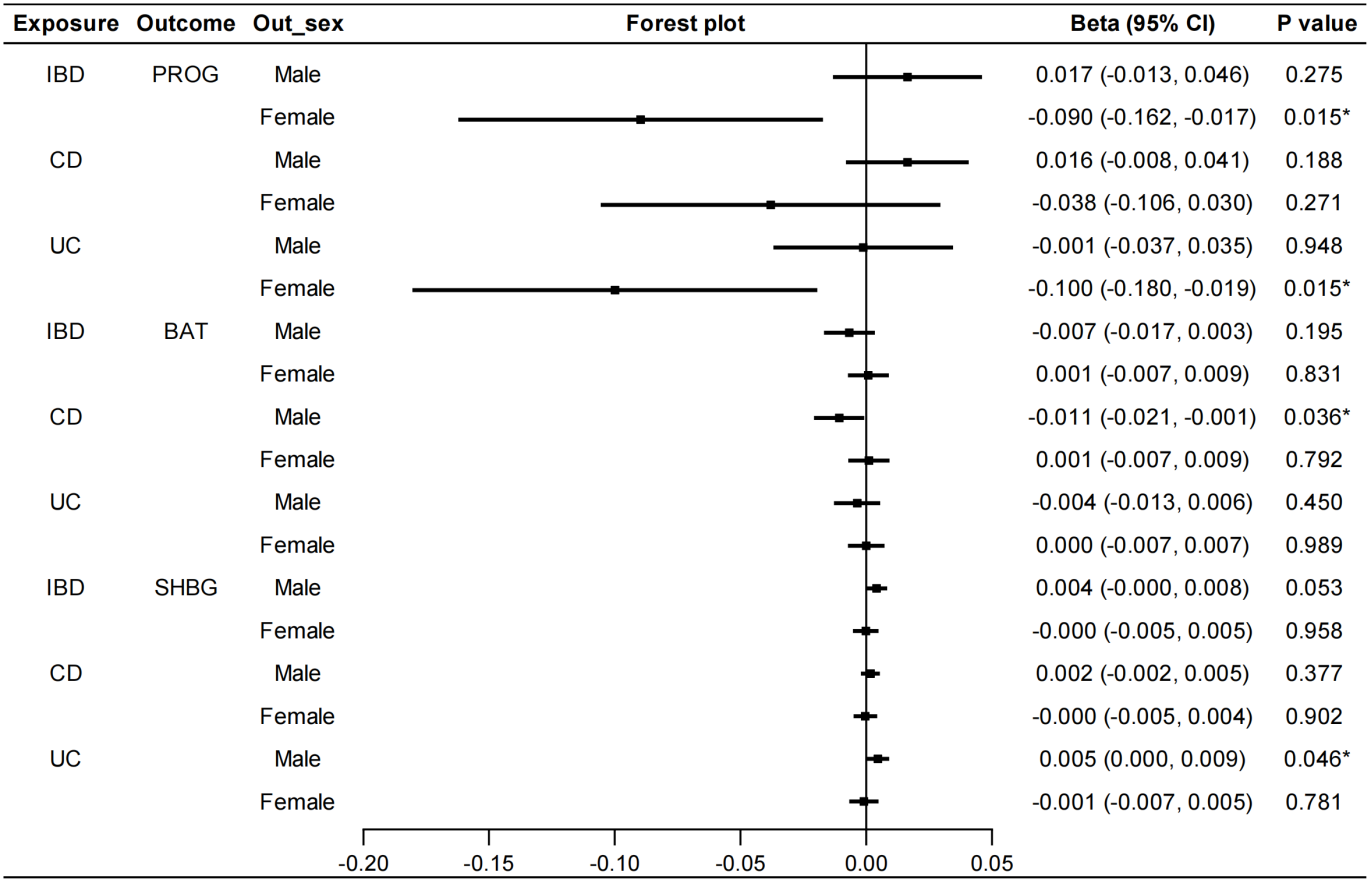
Sex-stratified causal effects of IBD/CD/UC on PROG, BAT and SHBG. Forest plots were used to show the MR estimate and 95% CI values using the inverse variance weighted method. *P<0.05. IBD, inflammatory bowel disease; CD, Crohn’s disease; UC, ulcerative colitis; PROG, progesterone; BAT, bioavailable testosterone; SHBG, sex hormone binding globulin; CI, confidence interval.

In the reverse MR analysis, we also conducted sex-stratified analyses to reveal genetically predicted gender-specific impact of IBD on sex hormone-associated markers. A negative correlation was found between UC and serum PROG levels in females (IVW: beta=-0.100, 95%CI: -0.180, -0.019, *P*=0.015) (**Figure 5**). No heterogeneity or pleiotropy was detected. UC was positively associated with serum SHBG levels in males (IVW: beta=0.005, 95%CI: 0.000, 0.009, *P*=0.046). Heterogeneity was also detected in this analysis (Cochran’s Q test: *P*<0.001), but MR-PRESSO analysis suggested that outliers did not influence the causal association (Outlier-corrected: *P*=0.030, Distortion test: *P*=0.447). The direction of MR-Egger regression and weighted median was consistent with that of IVW method in the above MR analyses. Furthermore, the IVW method revealed a significant association between CD and lower serum BAT levels in males (beta=-0.011, 95%CI: -0.021, -0.001, *P*=0.036). However, the direction of the results obtained from the MR-Egger method was contrary (beta=0.010, 95%CI: -0.013, 0.032, *P*=0.401), therefore, a stable causal association conclusion cannot be drawn. No causal effects of IBD on E2, TT and AMH were found (**Supplementary Figure S16**). Details of the causal analysis and sensitive analysis were shown in **Supplementary Table S17-S23**. The scatter plots of causal relationships were shown in **Supplementary Figures S17-S22**.

## 4 Discussion

IBD has become a significant global health issue because of the continuously increasing incidence, complex pathogenesis, and unsatisfactory treatment efficacy (43). Genetic susceptibility, immune disorders, intestinal dysbiosis, and environmental risk factors are associated with IBD (44). Sex hormones, which extensively distribute throughout the body, are involved in not only reproductive system disorders but also many immune-related diseases such as IBD (4). Studies have reported the modulatory effects of sex hormones on various biological functions of IBD, including intestinal barrier, mucosal immune activation and gut microbiota (4). However, the exact relationship between these sex hormones and IBD is unclear. In this two-sample MR analysis, we investigated the causal associations between sex hormones, sex hormone receptors, SHBG and IBD, based on multiple large-scale GWAS data. Our analyses revealed a negative correlation between serum E2 and BAT levels and the incidence of CD. We further conducted sex-stratified MR analysis, demonstrating a protective effect of E2 in males against CD and a protective effect of BAT in females against both CD and UC. Furthermore, we observed a negative correlation between serum SHBG levels and the incidence of CD in males. In the reverse MR analysis, CD was found to decrease the level of ER in the plasma of patients. UC was negatively correlated with PROG in females and was positively correlated with serum SHBG levels in males. No causal association was found between FSH, LH, PRL, PRLR, and AMH with IBD in this study.

As the primary sex hormone in females, estrogen has been found to balance cell proliferation, differentiation, and apoptosis by acting on ERs and the G protein-coupled estrogen receptor located on intestinal epithelial cells (4). Epidemiological studies on the association between estrogen and the incidence of IBD have predominantly been conducted in female populations. These studies primarily focus on comparing the differences in IBD incidence and development between women who use oral contraceptives or receive hormone replacement therapy and those who do not undergo exogenous estrogen treatment. However, due to the specific indication of hormone treatment, it is less feasible to conduct randomized controlled trials investigating the effects of hormone therapy on IBD. MR studies, with its advantage of random allocation, can effectively minimize various biases and balance confounding factors to a great extent, thus yielding higher levels of evidence compared to observational studies. In this MR study, we revealed a protective effect of E2 on CD. Importantly, we first reported the protective effect of E2 against CD in males rather than females. This interesting finding is consistent with the conclusion drawn from a previous study, which observed lower serum levels of E2 in male patients with CD compared to healthy individuals (13). Furthermore, Goodman et al. used SAMP1/YitFc mice that spontaneously develop CD-like symptoms and found that exogenous E2 administration suppressed intestinal inflammation in male mice but not in female mice, which may be because the immunoprotective effects mediated by distinct ER isoforms were impaired in T cells from SAMP-Female mice (45). In summary, our study supported for the protective role of E2 in males against CD.

Testosterone exists in two distinct forms in human body: free testosterone and conjugated testosterone. Free testosterone and testosterone bound to albumin, due to their ability to directly exert physiological effects, are referred to as bioavailable testosterone. Khalili et al. conducted a nested case-control study and discovered a correlation between pre-diagnostic total circulating testosterone levels in women and a lower risk of developing CD (46). However, this correlation was not observed in UC patients. In male patients with UC, there exists an inverse correlation between testosterone levels and the severity of rectal bleeding (12). Furthermore, male patients with CD exhibit lower serum testosterone levels compared to healthy individuals (13). In our study, although no association was discovered between male testosterone and CD or UC, we did observe a protective effect of female TT against CD but not UC, which was consistent with the previous finding (46). However, as a more meaningful marker, BAT in females had a protective effect against both CD and UC, which has not been reported before. In conclusion, we have discovered the protective effect of testosterone on both CD and UC, but only in females.

This MR study also showed the influence of IBD on sex hormones and their associated proteins. Previous studies have revealed that female IBD patients often experience delayed puberty onset and menstrual cycle irregularities (6). Both male and female IBD patients have a higher proportion of sexual and reproductive dysfunction compared to the general population (11, 47, 48). It is still uncertain whether changes of hormone levels mediate the effects of IBD on secondary sexual development, sexual function and reproductive function. In this study, we reported that UC decreased the level of PROG in females. PROG plays a vital role in maintaining normal reproductive function in females, and the lower PROG levels may contribute to the decline in reproductive function (49). Besides, our study identified that ER levels were reduced by CD. Luo et al. found that elevated levels of serum ERα were associated with male infertility (50). Thus, in light of our findings, this suggests that circulating ER may not mediate the effects of IBD on sexual and reproductive functions. Moreover, the two subtypes of ER, ERα and ERβ, may be differently associated with IBD, as a strong association was found between a low ERβ/ERα ratio and CD clinical and endoscopic activity (51). However, the relationship between IBD and ER subtypes cannot be analyzed using MR at present due to lack of GWAS data.

Additionally, we firstly reported the protective effect of male serum SHBG against CD. Interestingly, similar to a previous finding, this protective effect of SHBG was not observed in females (46). Furthermore, we found that UC was positively associated with serum SHBG levels in males. SHBG is a hepatically synthesized circulating steroid-binding protein that primarily binds to testosterone and estradiol in the bloodstream, regulating their concentrations. Currently, SHBG is considered an important biomarker for metabolic syndrome and hepatic steatosis, with potential therapeutic implications for various metabolic disorders (52). A low serum SHBG level has been observed as an inflammatory marker (53), suggesting a protective role of SHBG in metabolic and inflammatory diseases. Metabolic dysregulation has been implicated in the pathogenesis of IBD (54), with patients with metabolic dysfunction-associated fatty liver disease having a higher risk of developing CD (55). These findings supported the protective role of SHBG that we have identified in the development of CD. Further research is needed to explore the specific mechanisms by which SHBG intervenes in the pathogenesis of IBD.

There were still several limitations in this study. Firstly, the study only included European populations, and the generalizability of the research findings to other racial/ethnic groups may be limited. Secondly, a few IVs were not identified in the outcome GWAS dataset, which might impact the reliability of causal inference. Proxy SNPs were not utilized in this study as there is currently no conclusive evidence to support the superiority of using proxy SNPs. Thirdly, there is currently a lack of sex-stratified GWAS data for IBD. Although the GWAS data of the exposure and outcome factors used in the two-sample MR studies are not entirely sex-stratified, it is still considered acceptable (56). Further analysis should be conducted once sex-stratified data on IBD becomes available. Furthermore, the absence of GWAS data on IBD disease activity currently hinders the possibility of utilizing MR analysis to investigate the association between sex hormones and IBD disease activity. Therefore, the conclusions of this study require validation through large-scale epidemiological studies.

In conclusion, this MR study investigated the causal association between sex hormones and IBD, which contributes to a better understanding of the pathogenesis of IBD and can explain the gender differences in IBD incidence. In the future, further clinical and basic research should be conducted to elucidate how sex hormones are involved in the pathogenesis of IBD and to explore their potential diagnostic and therapeutic value in IBD.

## Data availability statement

The original contributions presented in the study are included in the article/**Supplementary Material**. Further inquiries can be directed to the corresponding author.

## Author contributions

FZ: Data curation, Investigation, Methodology, Software, Validation, Visualization, Writing – original draft. YH: Data curation, Investigation, Methodology, Software, Validation, Visualization, Writing – original draft. MX: Methodology, Software, Writing – review & editing. SW: Data curation, Software, Writing – review & editing. ZW: Data curation, Software, Writing – review & editing. FD: Conceptualization, Funding acquisition, Supervision, Writing – review & editing.
