# Supplementary material for: Associations between sex hormones, receptors, binding proteins and inflammatory bowel disease: a Mendelian randomization study
Source: Front Endocrinol (Lausanne). 2024 Apr 10;15:1272746. doi: 10.3389/fendo.2024.1272746 (PMC11039946; doi:10.3389/fendo.2024.1272746)

Supplementary figures for

**Associations between Sex Hormones, Receptors, Binding Proteins and Inflammatory Bowel Disease: A Mendelian Randomization Study.**

**Figure S1.** Causal effects of FSH, LH, PROG, PRL and BAT on IBD/CD/UC.

**Figure S2.** Scatter plots from sex hormones on IBD/CD/UC.

**Figure S3.** Causal effects of ER, PRLR and SHBG on IBD/CD/UC.

**Figure S4.** Scatter plots from ER/PRLR/SHBG on IBD/CD/UC.

**Figure S5.** Sex-stratified causal effects of PROG and AMH on IBD/CD/UC.

**Figure S6.** Scatter plots from sex-stratified E2 on IBD/CD/UC.

**Figure S7.** Scatter plots from sex-stratified PROG on IBD/CD/UC.

**Figure S8.** Scatter plots from sex-stratified BAT on IBD/CD/UC.

**Figure S9.** Scatter plots from sex-stratified TT on IBD/CD/UC.

**Figure S10.** Scatter plots from sex-stratified SHBG on IBD/CD/UC.

**Figure S11.** Scatter plots from AMH on IBD/CD/UC.

**Figure S12.** Causal effects of IBD/CD/UC on sex hormones.

**Figure S13.** Causal effects of IBD/CD/UC on PRLR and SHBG.

**Figure S14.** Scatter plots from IBD/CD/UC on sex hormones.

**Figure S15.** Scatter plots from IBD/CD/UC on ER/PRLR/SHBG.

**Figure S16.** Sex-stratified causal effects of IBD/CD/UC on E2, TT and AMH.

**Figure S17.** Scatter plots from IBD/CD/UC on sex-stratified E2.

**Figure S18.** Scatter plots from IBD/CD/UC on sex-stratified PROG.

**Figure S19.** Scatter plots from IBD/CD/UC on sex-stratified BAT.

**Figure S20.** Scatter plots from IBD/CD/UC on sex-stratified TT.

**Figure S21.** Scatter plots from IBD/CD/UC on sex-stratified SHBG.

**Figure S22.** Scatter plots from IBD/CD/UC on AMH.

**Figure S1. Causal effects of FSH, LH, PROG, PRL and BAT on IBD/CD/UC.**

Forest plots were used to show the MR estimate and 95% CI values using the inverse variance weighted method. Abbreviations: FSH, follicle-stimulating hormone; LH, luteinizing hormone; PROG, progesterone; PRL, prolactin; BAT, bioavailable testosterone; IBD, inflammatory bowel disease; CD, Crohn’s disease; UC, ulcerative colitis; OR, odds ratio; CI, confidence interval.


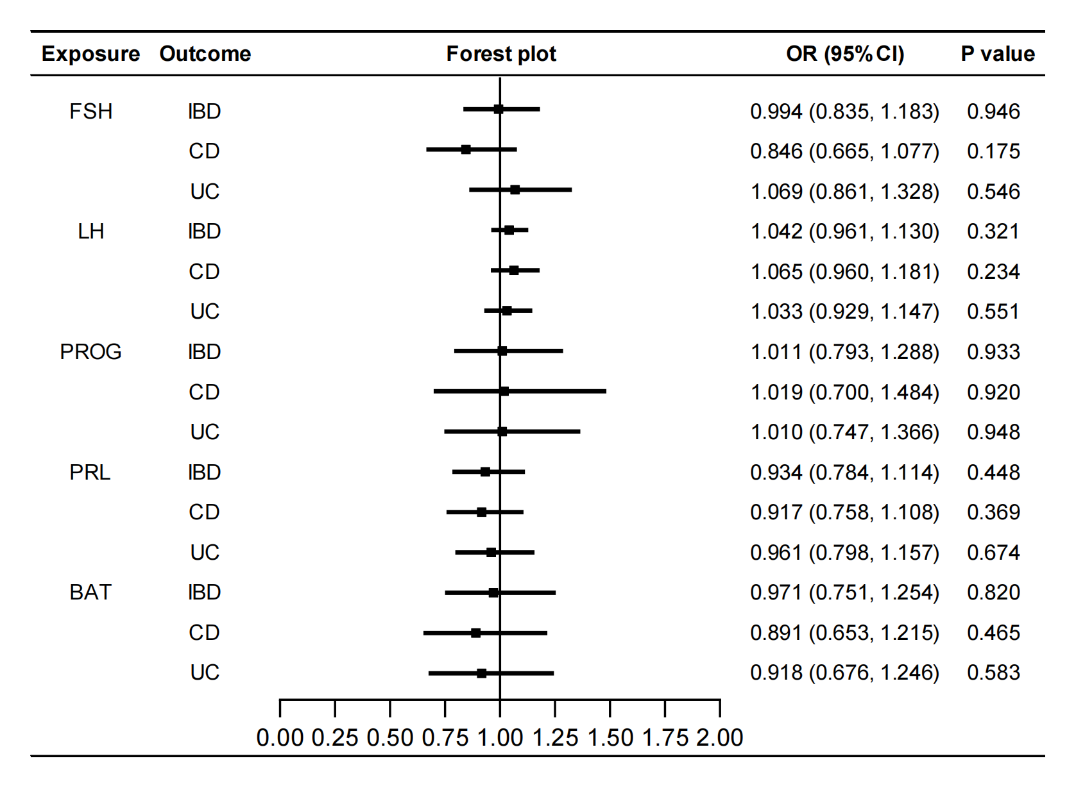


**Figure S2. Scatter plots from sex hormones on IBD/CD/UC.**

(A-C) Scatter plots from genetically predicted FSH on IBD/CD/UC; (D-F) Scatter plots from genetically predicted LH on IBD/CD/UC; (G-I) Scatter plots from genetically predicted E2 on IBD/CD/UC; (J-L) Scatter plots from genetically predicted PROG on IBD/CD/UC; (M-O) Scatter plots from genetically predicted PRL on IBD/CD/UC; (P-R) Scatter plots from genetically predicted BAT on IBD/CD/UC; (S-U) Scatter plots from genetically predicted TT on IBD/CD/UC. Abbreviations: FSH, follicle-stimulating hormone; LH, luteinizing hormone; E2, estradiol; PROG, progesterone; PRL, prolactin; BAT, bioavailable testosterone; TT, total testosterone; IBD, inflammatory bowel disease; CD, Crohn’s disease; UC, ulcerative colitis.


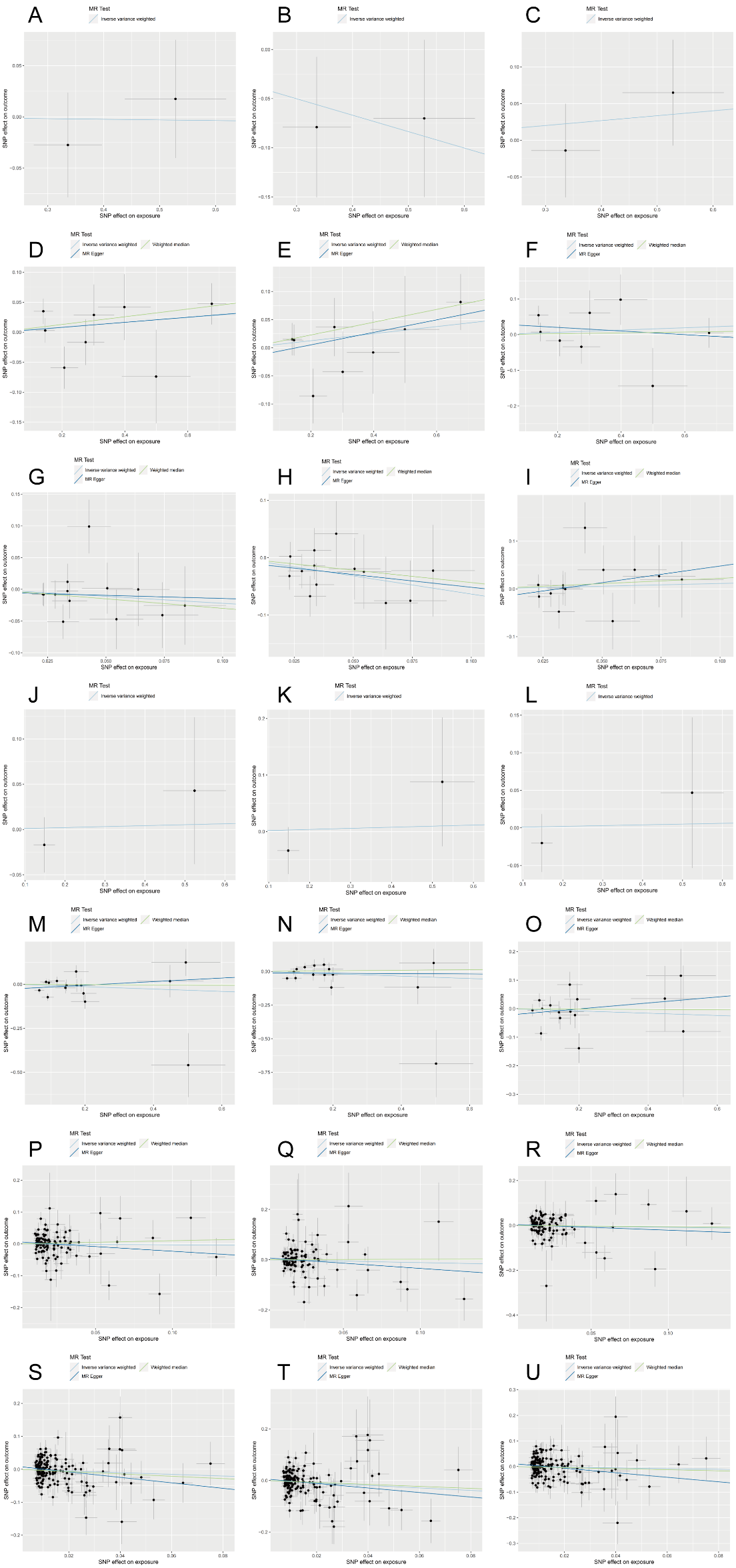


**Figure S3 Causal effects of ER, PRLR and SHBG on IBD/CD/UC.**

Forest plots were used to show the MR estimate and 95% CI values using the inverse variance weighted method. Abbreviations: ER, estrogen receptor; PRLR, prolactin receptor; SHBG, sex hormone binding globulin; IBD, inflammatory bowel disease; CD, Crohn’s disease; UC, ulcerative colitis; OR, odds ratio; CI, confidence interval.


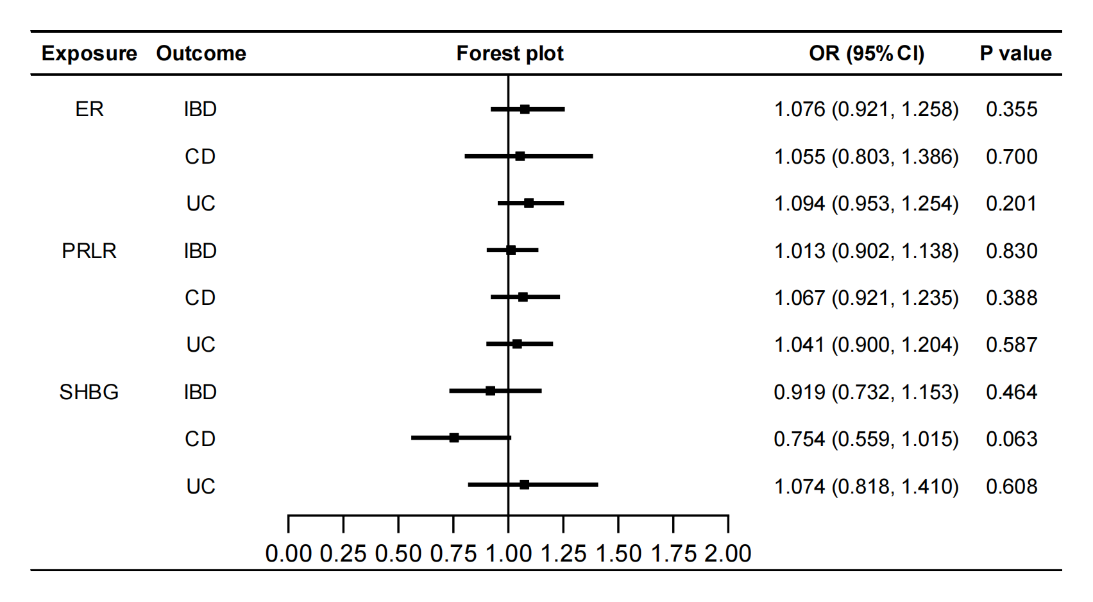


**Figure S4. Scatter plots from ER/PRLR/SHBG on IBD/CD/UC.**

(A-C) Scatter plots from genetically predicted ER on IBD/CD/UC; (D-F) Scatter plots from genetically predicted PRLR on IBD/CD/UC; (G-I) Scatter plots from genetically predicted SHBG on IBD/CD/UC. Abbreviations: ER, estrogen receptor; PRLR, prolactin receptor; SHBG, sex hormone binding globulin; IBD, inflammatory bowel disease; CD, Crohn’s disease; UC, ulcerative colitis.


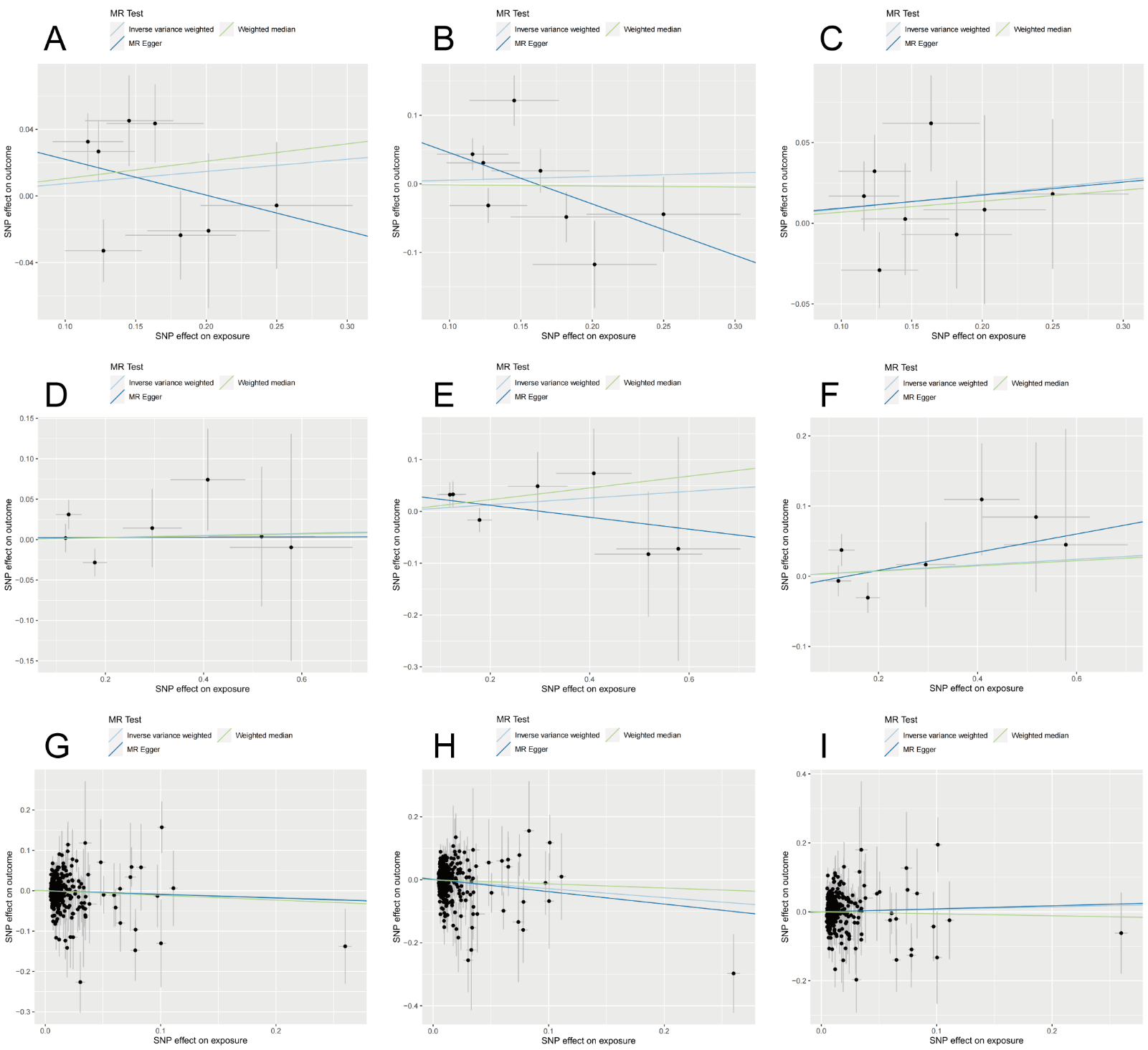


**Figure S5. Sex-stratified causal effects of PROG and AMH on IBD/CD/UC.**

Forest plots were used to show the MR estimate and 95% CI values using the inverse variance weighted method. Abbreviations: PROG, progesterone; AMH, anti-Müllerian hormone; IBD, inflammatory bowel disease; CD, Crohn’s disease; UC, ulcerative colitis; OR, odds ratio; CI, confidence interval.


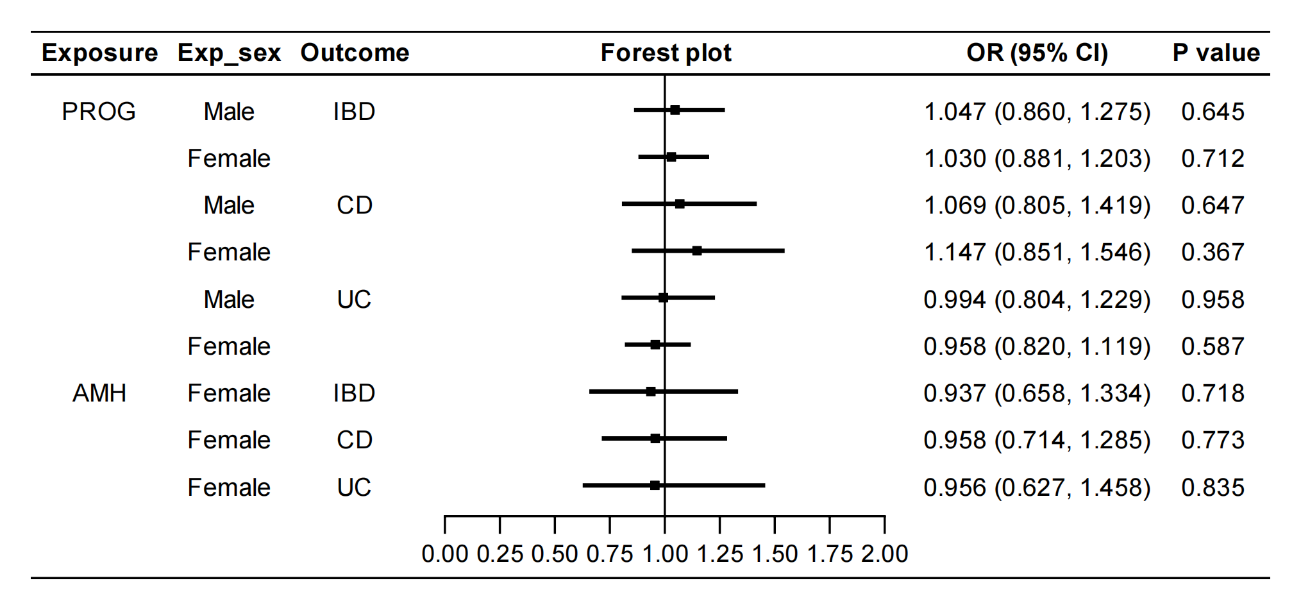


**Figure S6. Scatter plots from sex-stratified E2 on IBD/CD/UC.**

(A-C) Scatter plots from genetically predicted E2 in males on IBD/CD/UC; (D-F) Scatter plots from genetically predicted E2 in females on IBD/CD/UC. Abbreviations: E2, estradiol; IBD, inflammatory bowel disease; CD, Crohn’s disease; UC, ulcerative colitis.


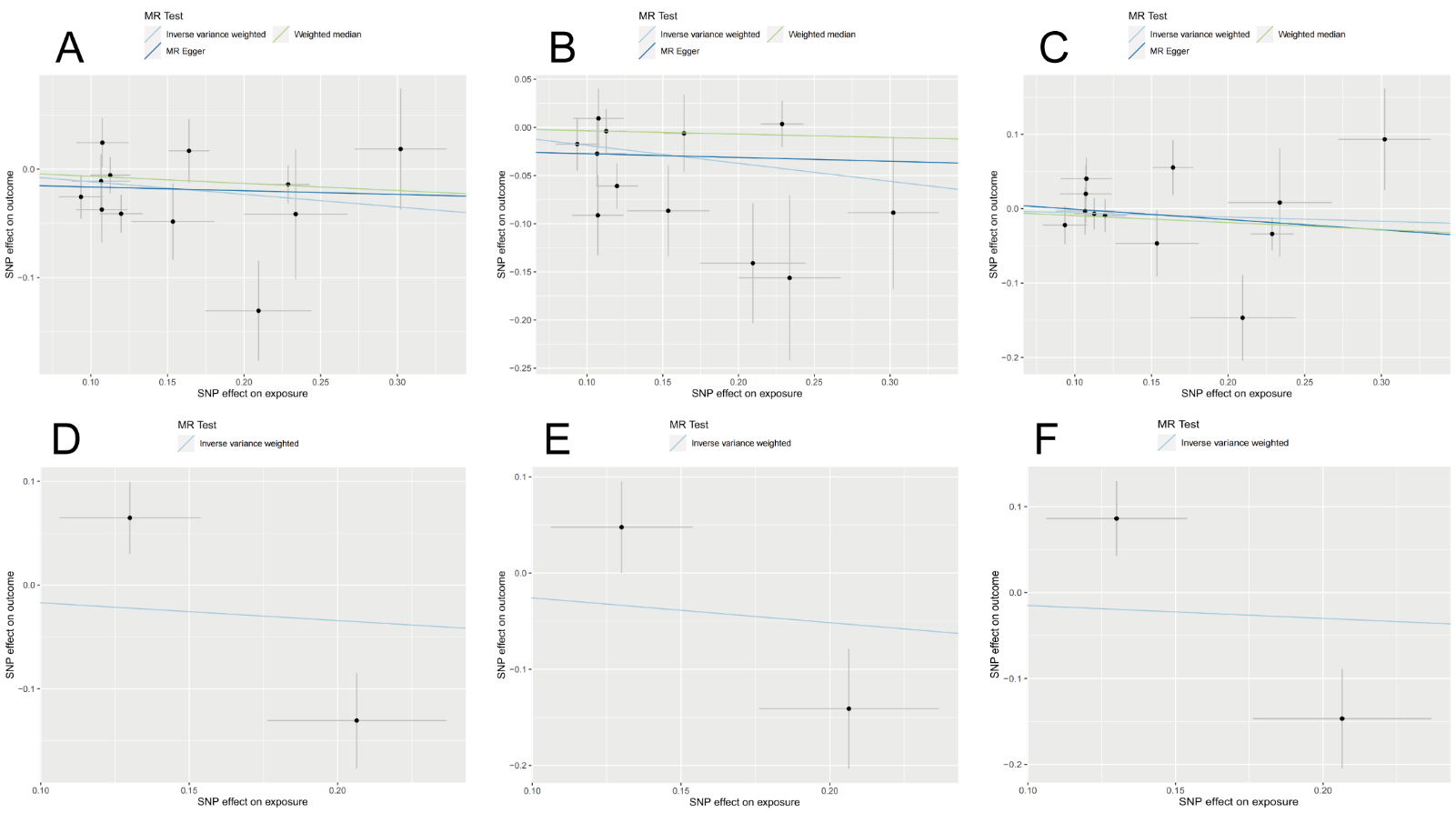


**Figure S7. Scatter plots from sex-stratified PROG on IBD/CD/UC.**

(A-C) Scatter plots from genetically predicted PROG in males on IBD/CD/UC; (D-F) Scatter plots from genetically predicted PROG in females on IBD/CD/UC. Abbreviations: PROG, progesterone; IBD, inflammatory bowel disease; CD, Crohn’s disease; UC, ulcerative colitis.


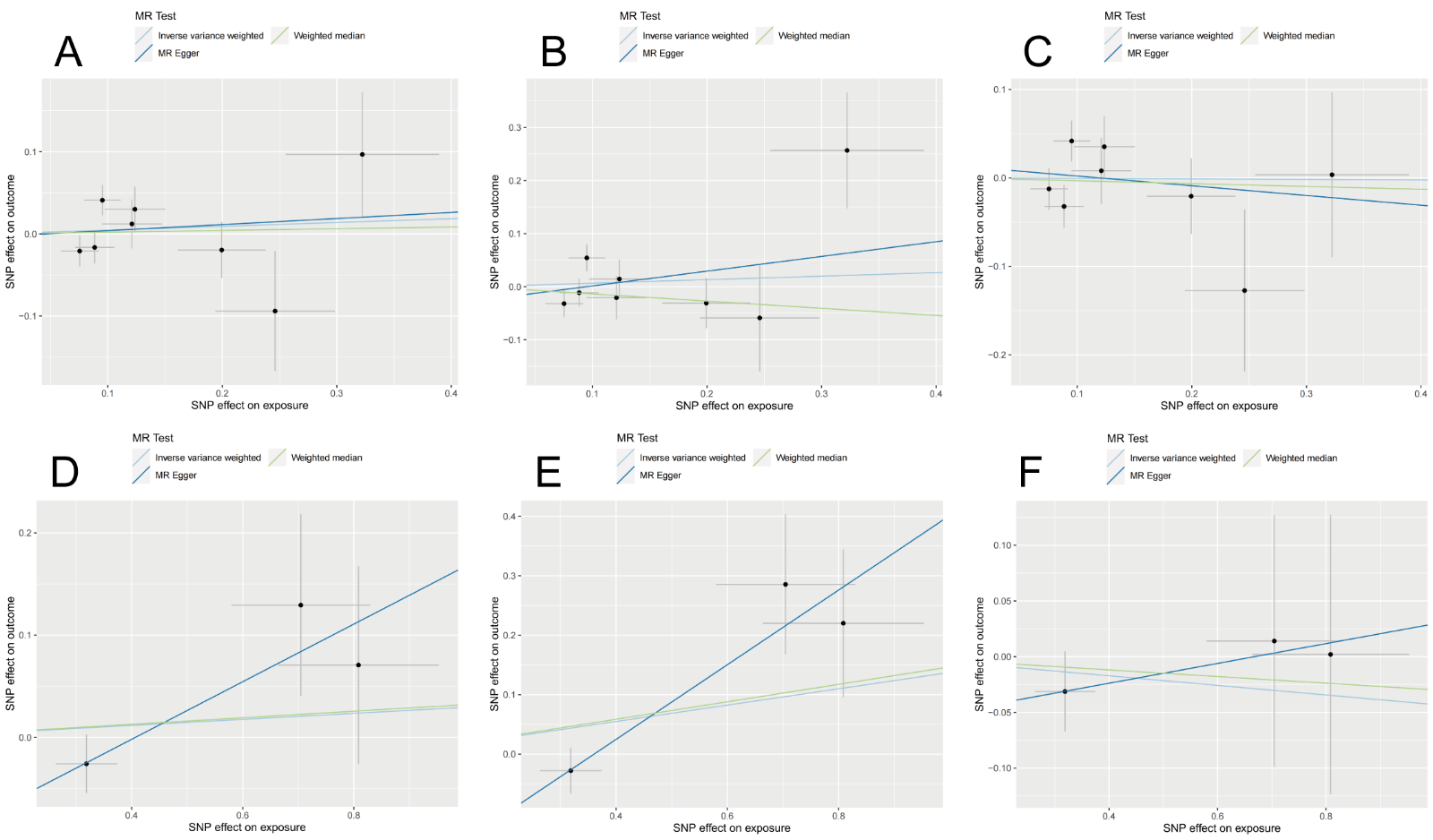


**Figure S8. Scatter plots from sex-stratified BAT on IBD/CD/UC.**

(A-C) Scatter plots from genetically predicted BAT in males on IBD/CD/UC; (D-F) Scatter plots from genetically predicted BAT in females on IBD/CD/UC. Abbreviations: BAT, bioavailable testosterone; IBD, inflammatory bowel disease; CD, Crohn’s disease; UC, ulcerative colitis.


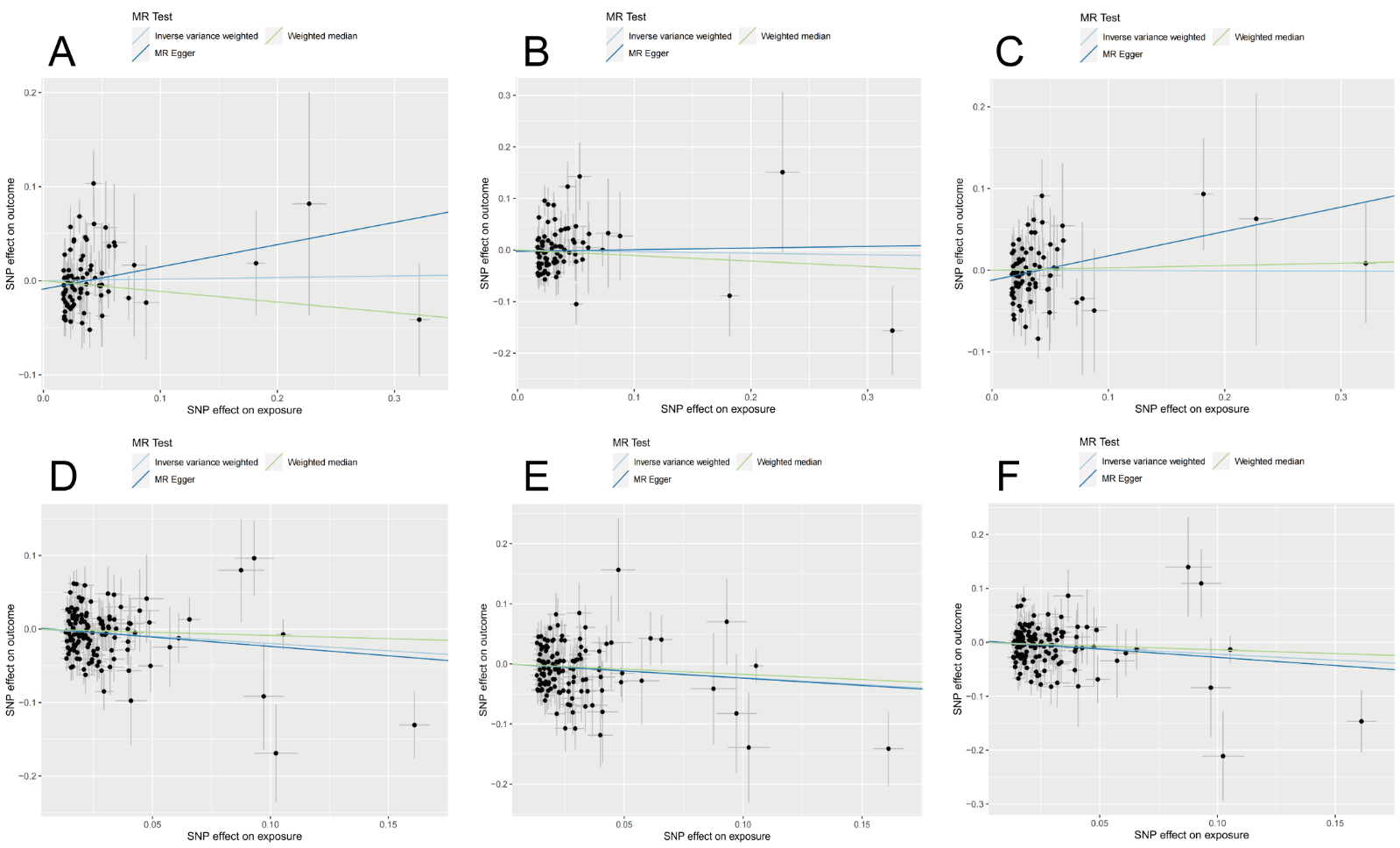


**Figure S9. Scatter plots from sex-stratified TT on IBD/CD/UC.**

(A-C) Scatter plots from genetically predicted TT in males on IBD/CD/UC; (D-F) Scatter plots from genetically predicted TT in females on IBD/CD/UC. Abbreviations: TT, total testosterone; IBD, inflammatory bowel disease; CD, Crohn’s disease; UC, ulcerative colitis.


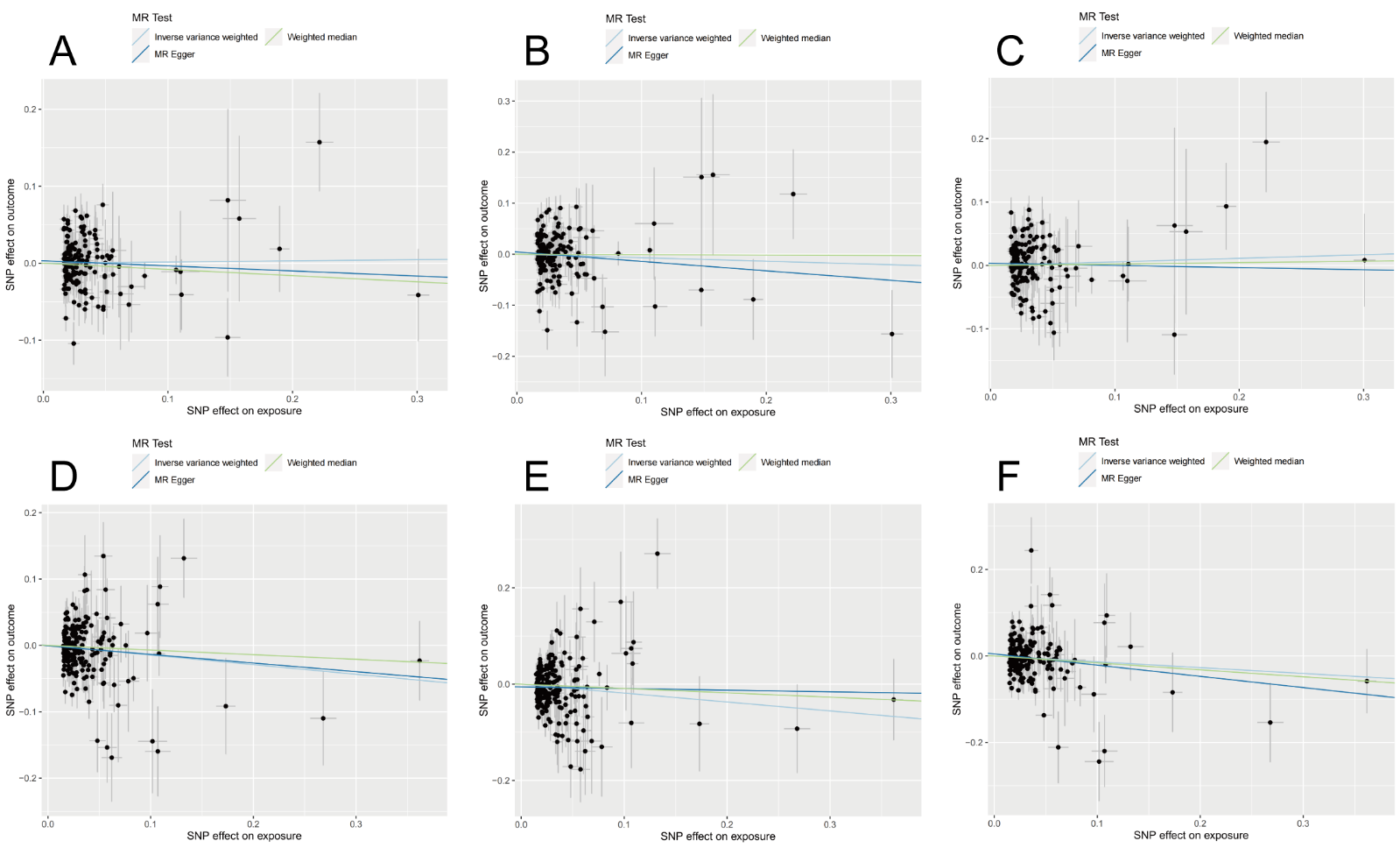


**Figure S10. Scatter plots from sex-stratified SHBG on IBD/CD/UC.**

(A-C) Scatter plots from genetically predicted SHBG in males on IBD/CD/UC; (D-F) Scatter plots from genetically predicted SHBG in females on IBD/CD/UC. Abbreviations: SHBG, sex hormone-binding globulin; IBD, inflammatory bowel disease; CD, Crohn’s disease; UC, ulcerative colitis.


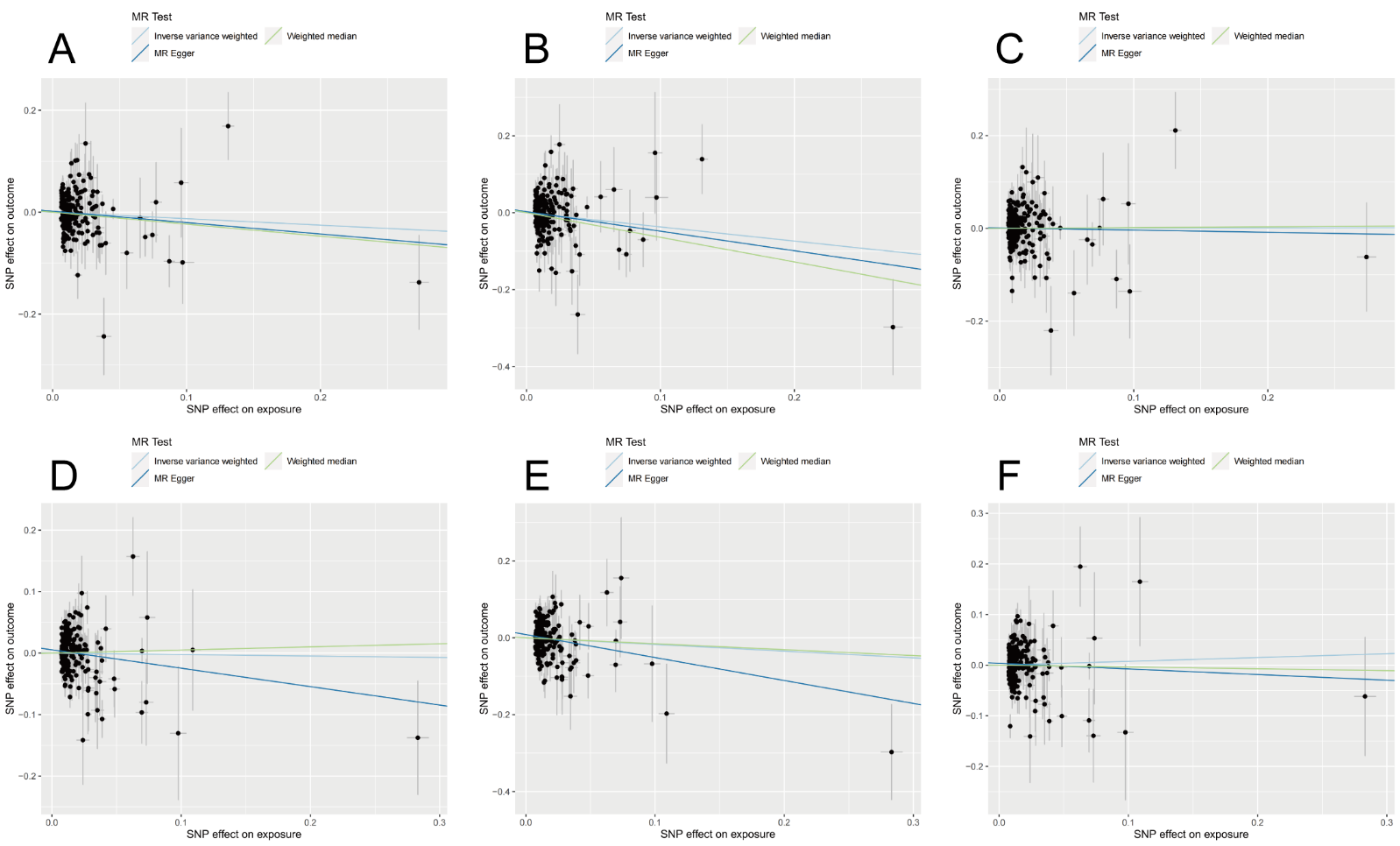


**Figure S11. Scatter plots from AMH on IBD/CD/UC.**

(A) Scatter plots from genetically predicted AMH in females on IBD; (B) Scatter plots from genetically predicted AMH in females on CD; (C) Scatter plots from genetically predicted AMH in females on UC. Abbreviations: AMH, anti-Müllerian hormone; IBD, inflammatory bowel disease; CD, Crohn’s disease; UC, ulcerative colitis.


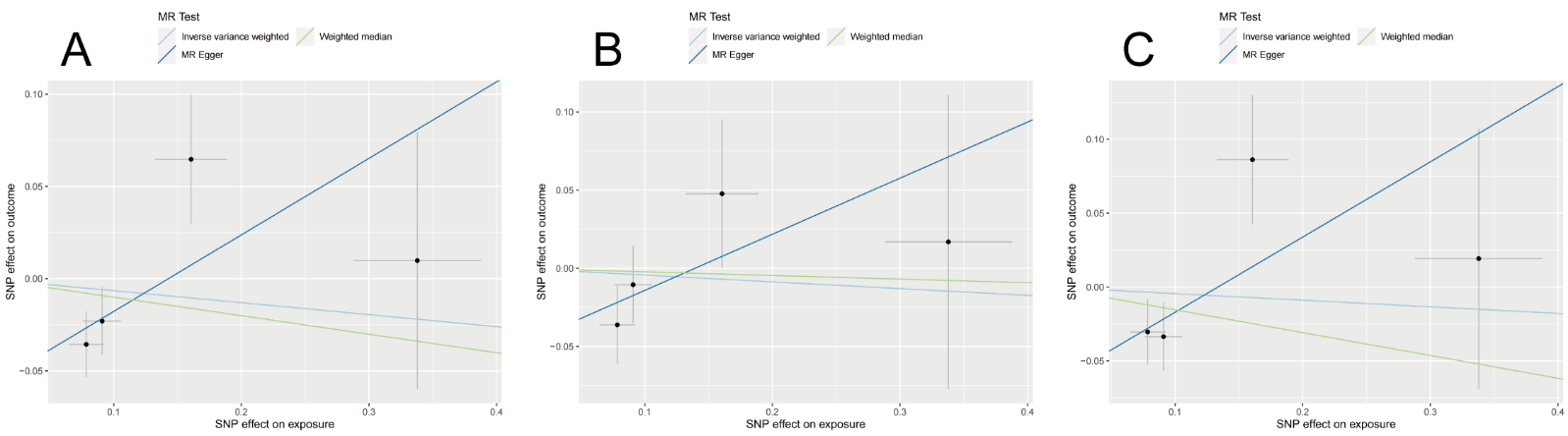


**Figure S12. Causal effects of IBD/CD/UC on sex hormones.**

Forest plots were used to show the MR estimate and 95% CI values using the inverse variance weighted method. Abbreviations: IBD, inflammatory bowel disease; CD, Crohn’s disease; UC, ulcerative colitis; FSH, follicle-stimulating hormone; LH, luteinizing hormone; E2, estradiol; PROG, progesterone; PRL, prolactin; BAT, bioavailable testosterone; TT, total testosterone; CI, confidence interval.


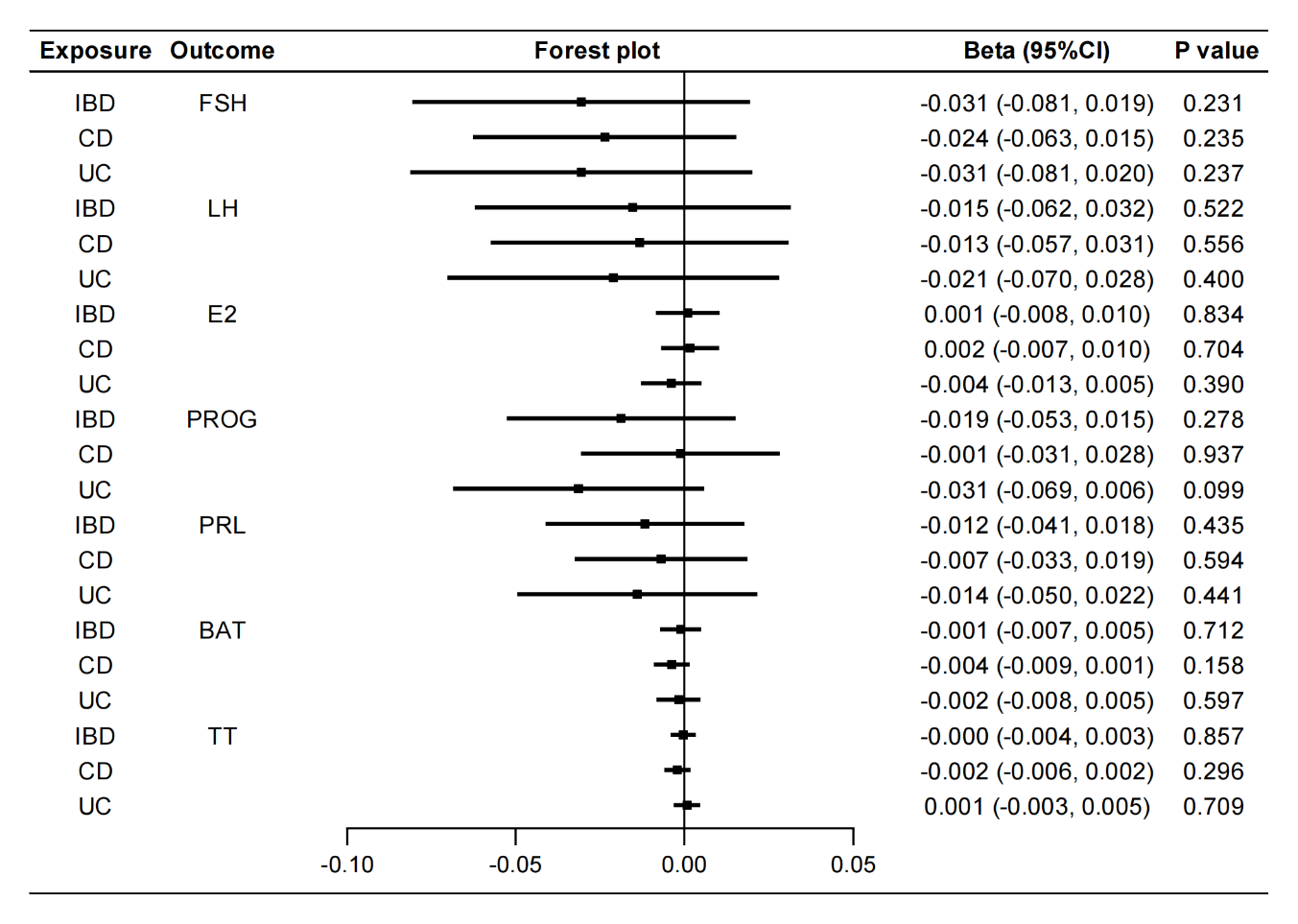


**Figure S13 Causal effects of IBD/CD/UC on PRLR and SHBG.**

Forest plots were used to show the MR estimate and 95% CI values using the inverse variance weighted method. Abbreviations: IBD, inflammatory bowel disease; CD, Crohn’s disease; UC, ulcerative colitis; PRLR, prolactin receptor; SHBG, sex hormone binding globulin; CI, confidence interval.


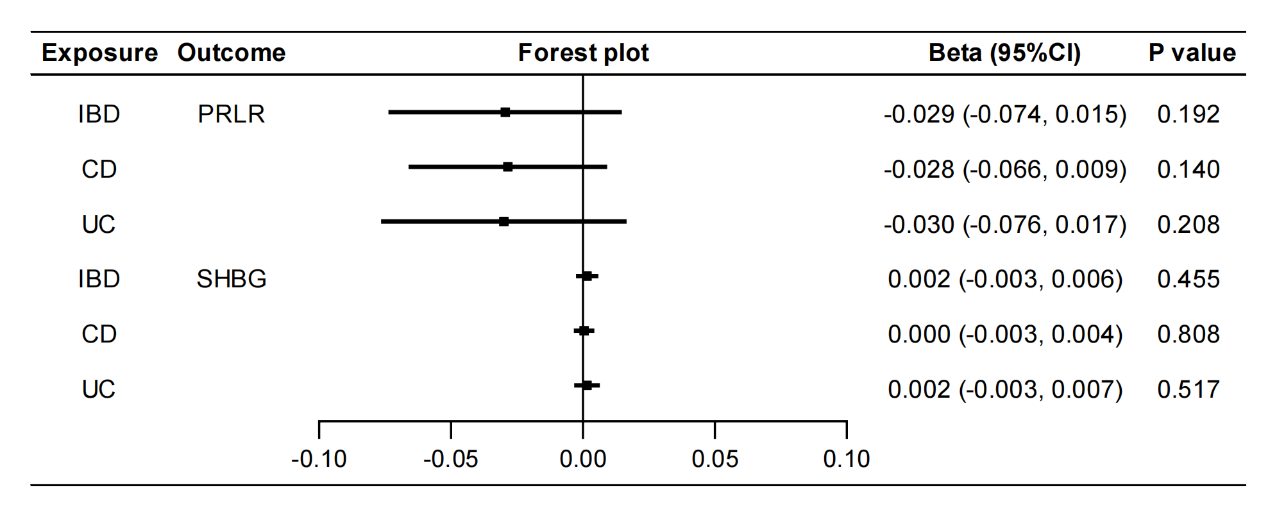


**Figure S14. Scatter plots from IBD/CD/UC on sex hormones.**

(A-C) Scatter plots from genetically predicted IBD/CD/UC on FSH; (D-F) Scatter plots from genetically predicted IBD/CD/UC on LH; (G-I) Scatter plots from genetically predicted IBD/CD/UC on E2; (J-L) Scatter plots from genetically predicted IBD/CD/UC on PROG; (M-O) Scatter plots from genetically predicted IBD/CD/UC on PRL; (P-R) Scatter plots from genetically predicted IBD/CD/UC on BAT; (S-U) Scatter plots from genetically predicted IBD/CD/UC on TT. Abbreviations: IBD, inflammatory bowel disease; CD, Crohn’s disease; UC, ulcerative colitis; FSH, follicle-stimulating hormone; LH, luteinizing hormone; E2, estradiol; PROG, progesterone; PRL, prolactin; BAT, bioavailable testosterone; TT, total testosterone.


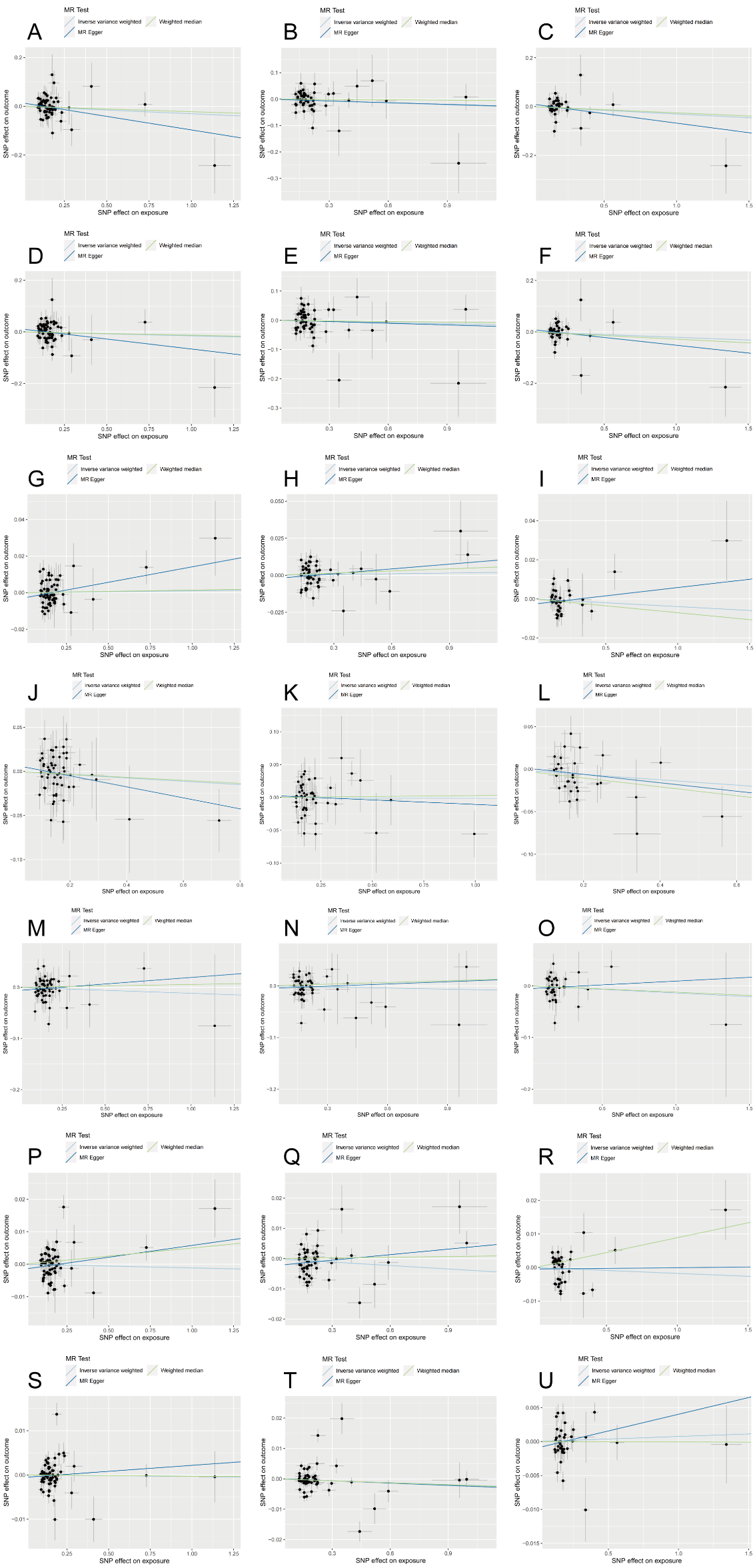


**Figure S15. Scatter plots from IBD/CD/UC on ER/PRLR/SHBG.**

(A-C) Scatter plots from genetically predicted IBD/CD/UC on ER; (D-F) Scatter plots from genetically predicted IBD/CD/UC on PRLR; (G-I) Scatter plots from genetically predicted IBD/CD/UC on SHBG. Abbreviations: IBD, inflammatory bowel disease; CD, Crohn’s disease; UC, ulcerative colitis; ER, estrogen receptor; PRLR, prolactin receptor; SHBG, sex hormone-binding globulin.


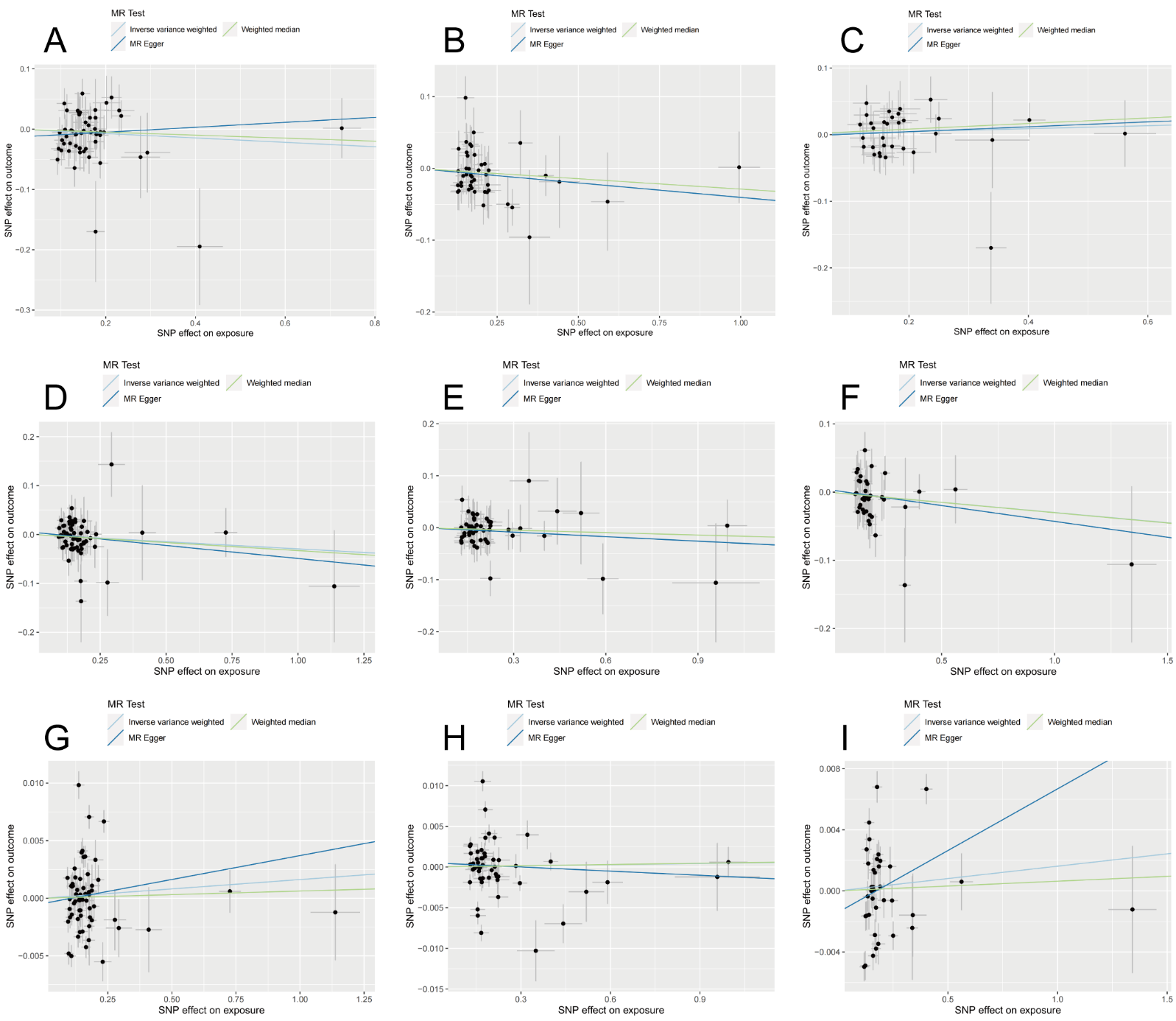


**Figure S16. Sex-stratified causal effects of IBD/CD/UC on E2, TT and AMH.**

Forest plots were used to show the MR estimate and 95% CI values using the inverse variance weighted method. Abbreviations: IBD, inflammatory bowel disease; CD, Crohn’s disease; UC, ulcerative colitis; E2, estradiol; TT, total testosterone; AMH, anti-Müllerian hormone; CI, confidence interval.


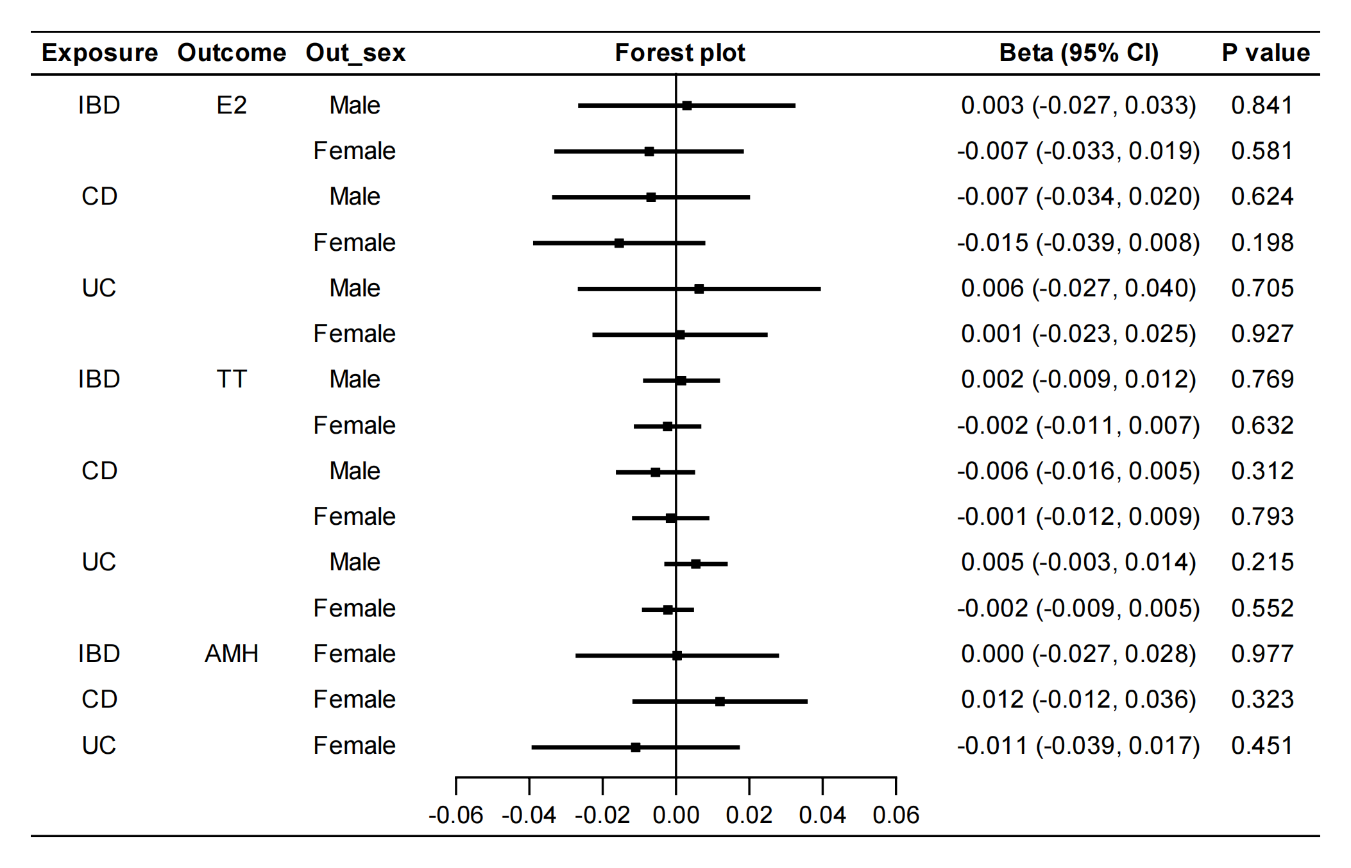


**Figure S17. Scatter plots from IBD/CD/UC on sex-stratified E2.**

(A-C) Scatter plots from genetically predicted IBD/CD/UC on E2 in males; (D-F) Scatter plots from genetically predicted IBD/CD/UC on E2 in females. Abbreviations: IBD, inflammatory bowel disease; CD, Crohn’s disease; UC, ulcerative colitis; E2, estradiol.


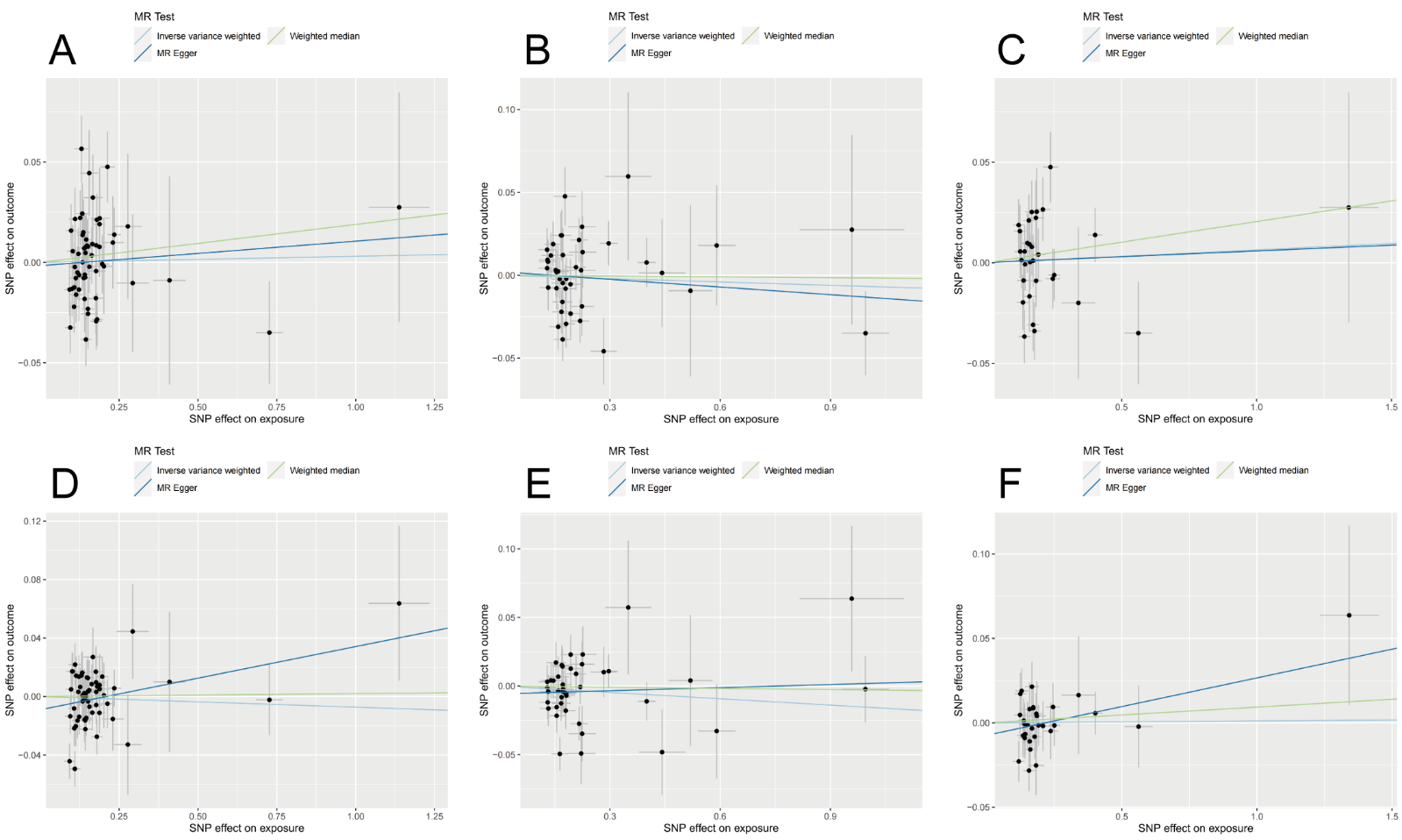


**Figure S18. Scatter plots from IBD/CD/UC on sex-stratified PROG.**

(A-C) Scatter plots from genetically predicted IBD/CD/UC on PROG in males; (D-F) Scatter plots from genetically predicted IBD/CD/UC on PROG in females. Abbreviations: IBD, inflammatory bowel disease; CD, Crohn’s disease; UC, ulcerative colitis; PROG, progesterone.


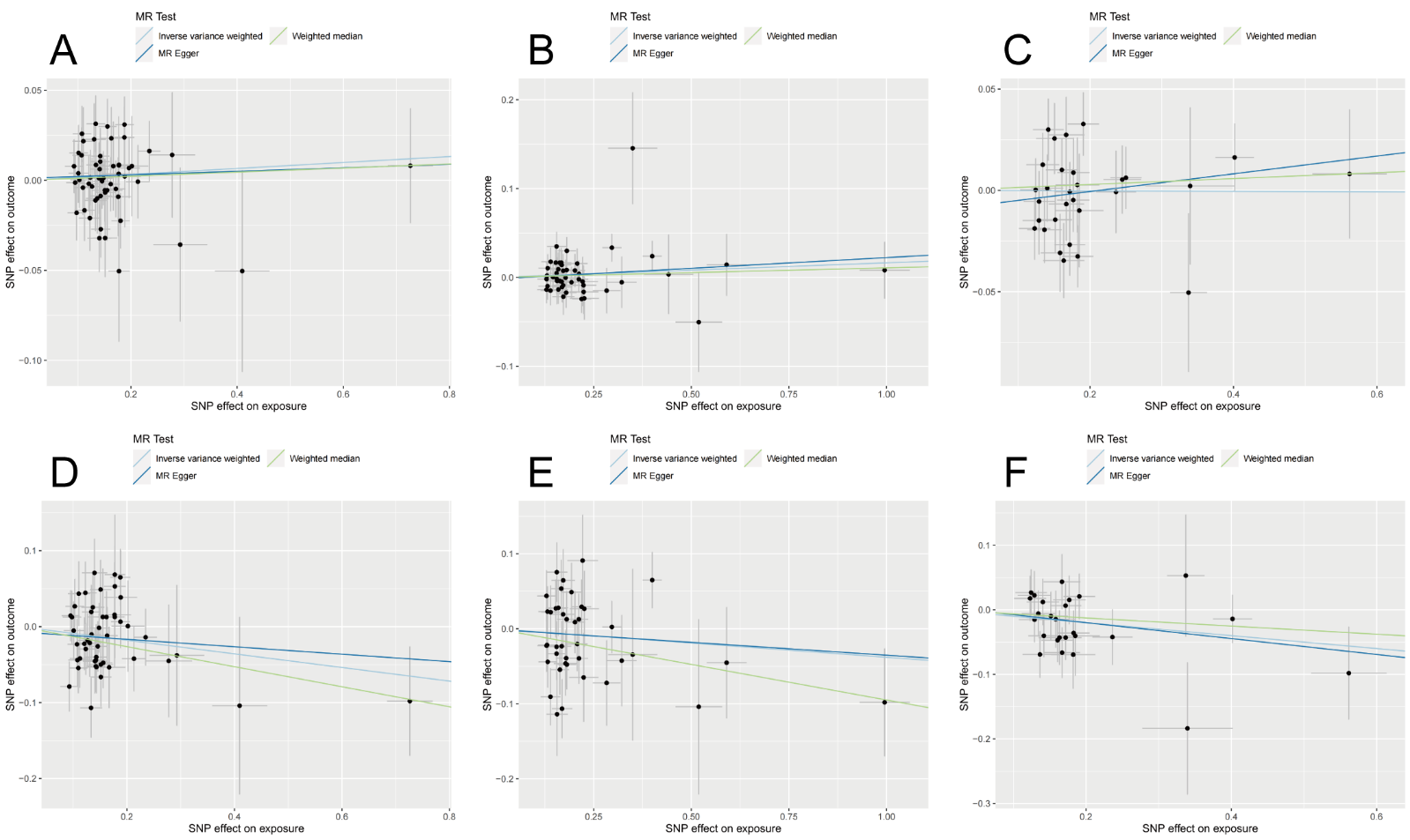


**Figure S19. Scatter plots from IBD/CD/UC on sex-stratified BAT.**

(A-C) Scatter plots from genetically predicted IBD/CD/UC on BAT in males; (D-F) Scatter plots from genetically predicted IBD/CD/UC on BAT in females. Abbreviations: IBD, inflammatory bowel disease; CD, Crohn’s disease; UC, ulcerative colitis; BAT, bioavailable testosterone.


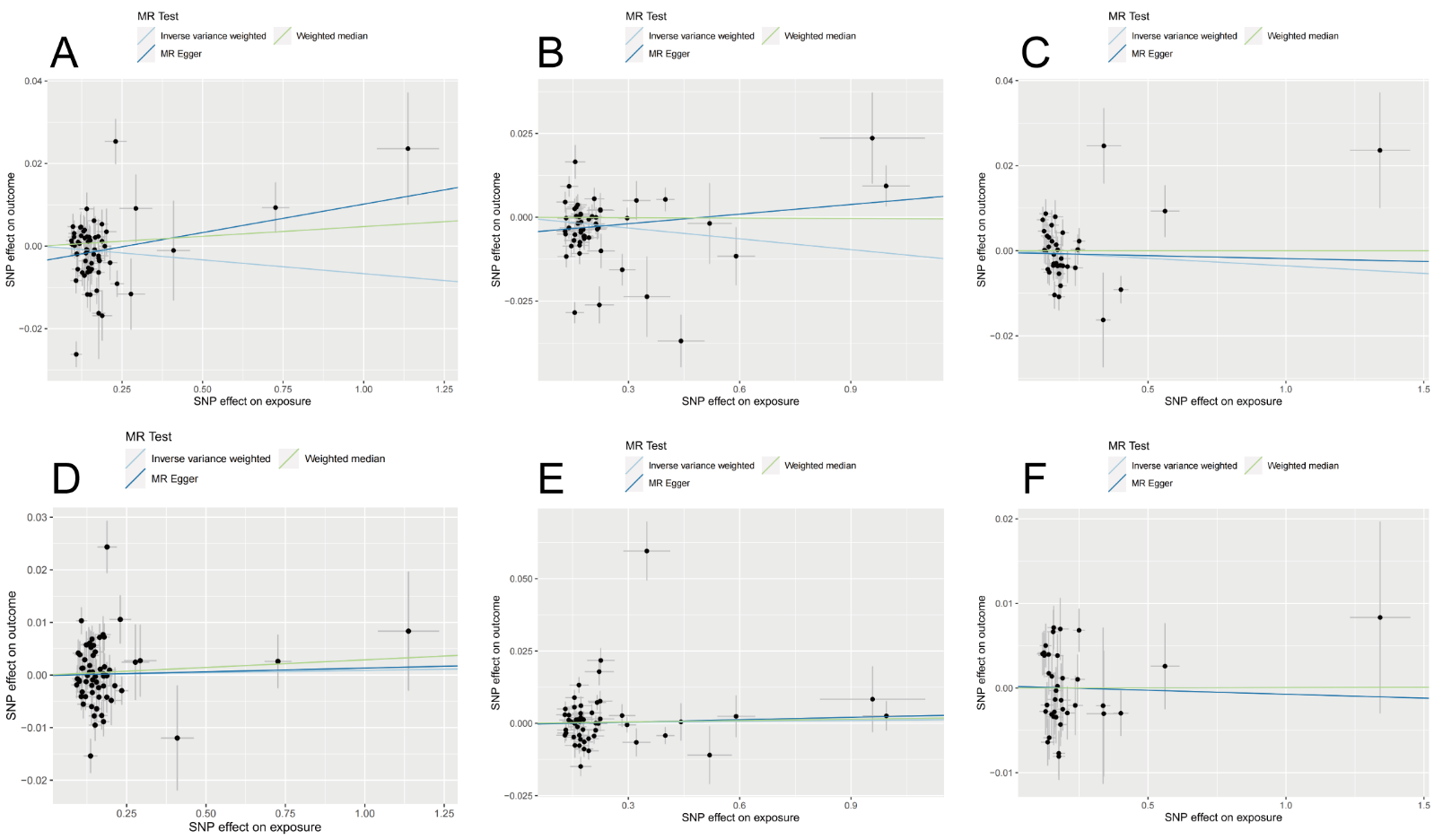
**Figure S20. Scatter plots from IBD/CD/UC on sex-stratified TT.**

(A-C) Scatter plots from genetically predicted IBD/CD/UC on TT in males; (D-F) Scatter plots from genetically predicted IBD/CD/UC on TT in females. Abbreviations: IBD, inflammatory bowel disease; CD, Crohn’s disease; UC, ulcerative colitis; TT, total testosterone.


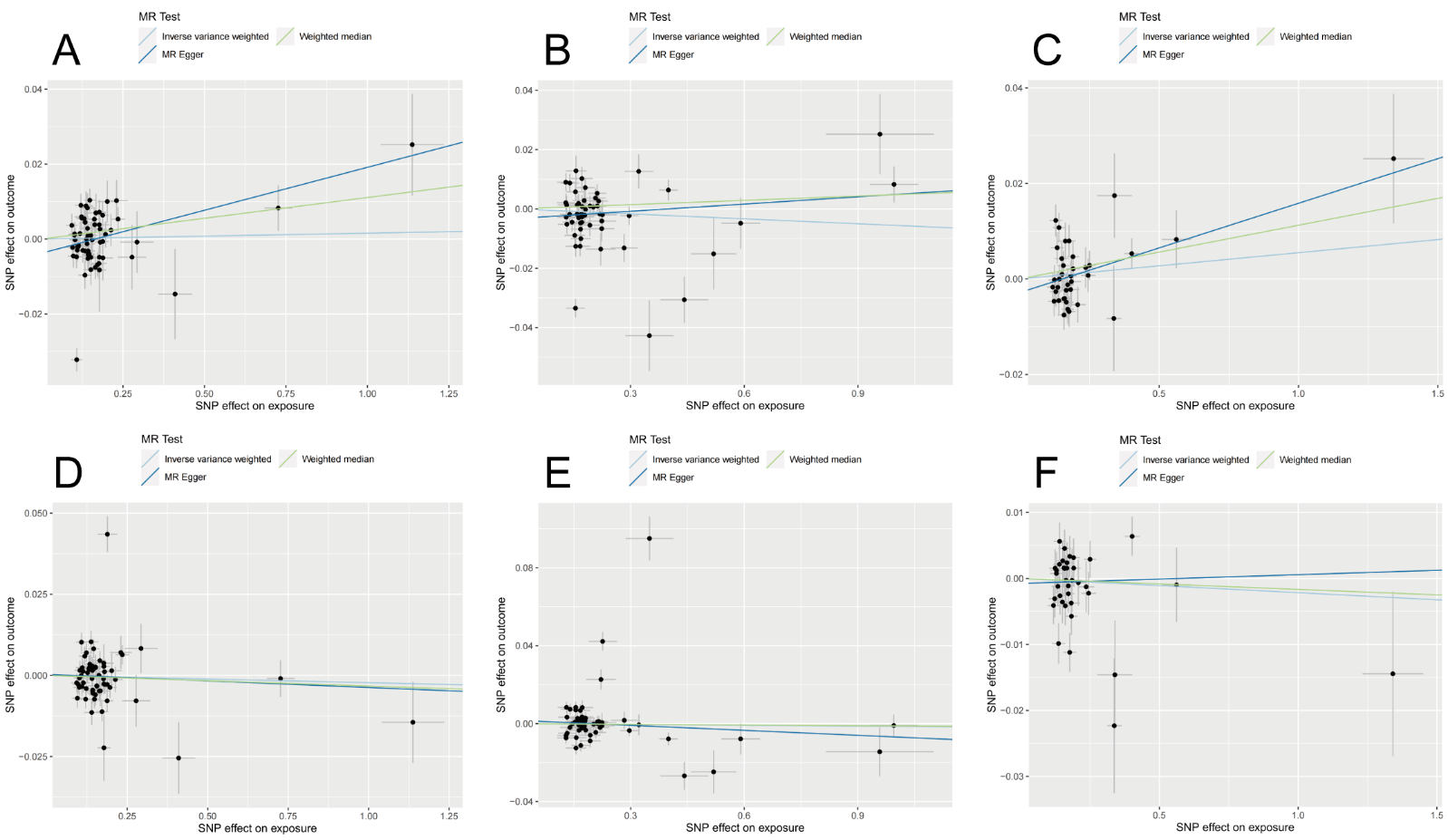


**Figure S21. Scatter plots from IBD/CD/UC on sex-stratified SHBG.**

(A-C) Scatter plots from genetically predicted IBD/CD/UC on SHBG in males; (D-F) Scatter plots from genetically predicted IBD/CD/UC on SHBG in females. Abbreviations: IBD, inflammatory bowel disease; CD, Crohn’s disease; UC, ulcerative colitis; SHBG, sex hormone-binding globulin.


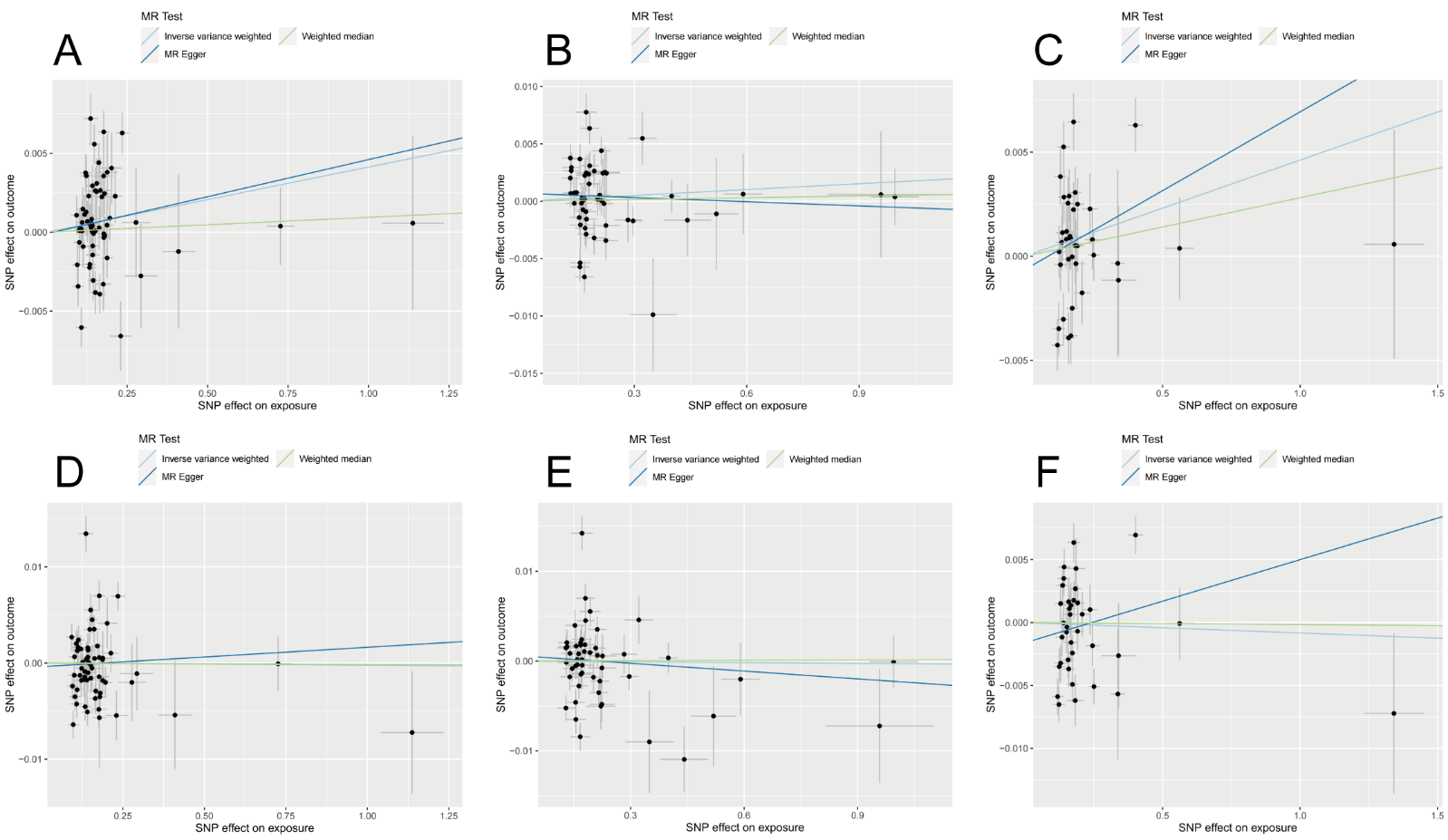


**Figure S22. Scatter plots from IBD/CD/UC on AMH.**

(A) Scatter plots from genetically predicted IBD on AMH in females; (B) Scatter plots from genetically predicted CD on AMH in females; (C) Scatter plots from genetically predicted UC on AMH in females. Abbreviations: IBD, inflammatory bowel disease; CD, Crohn’s disease; UC, ulcerative colitis; AMH, anti-Müllerian hormone.


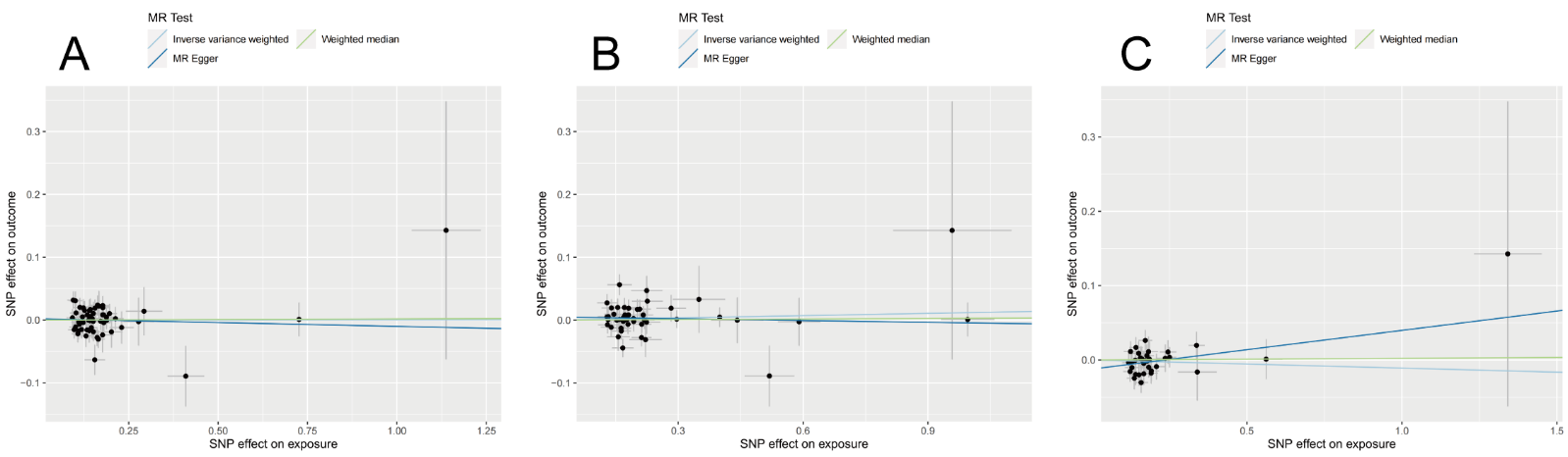

Supplement: Supplementary file 1 [file DataSheet_1.docx]
