# Supplementary material for: Associations between sex hormones, receptors, binding proteins and inflammatory bowel disease: a Mendelian randomization study
Source: Front Endocrinol (Lausanne). 2024 Apr 10;15:1272746. doi: 10.3389/fendo.2024.1272746 (PMC11039946; doi:10.3389/fendo.2024.1272746)
Supplement: Supplementary file 2 [file DataSheet_2.docx]

Supplementary tables for

**Associations between Sex Hormones, Receptors, Binding Proteins and Inflammatory Bowel Disease: A Mendelian Randomization Study.**

**Table S1.** The characteristics of GWAS studies.

**Table S2.** Heterogeneity and pleiotropy tests for the associations between sex hormones and IBD/CD/UC.

**Table S3.** Causal effects of sex hormones on IBD/CD/UC using different models.

**Table S4.** Causal effects of ER, PRLR and SHBG on IBD/CD/UC using different models.

**Table S5.** Heterogeneity and pleiotropy tests for the associations between ER/PRLR/SHBG and IBD/CD/UC.

**Table S6.** Heterogeneity and pleiotropy tests for the sex-stratified associations between E2/PROG/BAT/TT/SHBG/AMH and IBD/CD/UC.

**Table S7.** Sex-stratified causal effects of E2 on IBD/CD/UC using different models.

**Table S8.** Sex-stratified causal effects of PROG on IBD/CD/UC using different models.

**Table S9.** Sex-stratified causal effects of BAT on IBD/CD/UC using different models.

**Table S10.** Sex-stratified causal effects of TT on IBD/CD/UC using different models.

**Table S11.** Sex-stratified causal effects of SHBG on IBD/CD/UC using different models.

**Table S12.** Causal effects of AMH in female on IBD/CD/UC using different models.

**Table S13.** Causal effects of IBD/CD/UC on sex hormones using different models.

**Table S14.** Heterogeneity and pleiotropy tests for the associations between sex hormones and IBD/CD/UC.

**Table S15.** Causal effects of IBD/CD/UC on ER, PRLR and SHBG using different models.

**Table S16.** Heterogeneity and pleiotropy tests for the associations between ER/PRLR/SHBG and IBD/CD/UC.

**Table S17.** Sex-stratified causal effects of IBD/CD/UC on E2 using different models.

**Table S18.** Sex-stratified causal effects of IBD/CD/UC on PROG using different models.

**Table S19.** Sex-stratified causal effects of IBD/CD/UC on BAT using different models.

**Table S20.** Sex-stratified causal effects of IBD/CD/UC on TT using different models.

**Table S21.** Sex-stratified causal effects of IBD/CD/UC on SHBG using different models.

**Table S22.** Sex-stratified causal effects of IBD/CD/UC on AMH using different models.

**Table S23.** Heterogeneity and pleiotropy tests for the sex-stratified associations between IBD/CD/UC and E2/PROG/BAT/TT/SHBG/AMH.

**Table S1. The characteristics of GWAS studies.**

| Traits | Sex | Data sources | Sample size  (cases/controls) | Ancestry | PMID | Numbers of IVs |
| --- | --- | --- | --- | --- | --- | --- |
| FSH | Both | IEU | 3,301 | EUR | 29875488 | 2 |
| LH | Both | IEU | 3,301 | EUR | 29875488 | 9 |
| E2 | Both | Pan UK Biobank | 67,623 | 93.7% EUR | / | 14 |
| E2 | Male | GWAS Catalog | 147,690 | EUR | 34255042 | 12 |
| E2 | Female | GWAS Catalog | 163,985 | EUR | 34255042 | 2 |
| PROG | Both | Leipzig Health Atlas | 2,070 | EUR | 31169883 | 2 |
| PROG | Male | Leipzig Health Atlas | 1,358 | EUR | 31169883 | 8 |
| PROG | Female | Leipzig Health Atlas | 1,261 | EUR | 31169883 | 3 |
| PRL | Both | GWAS Catalog | 30,931 | EUR | 33067605 | 15 |
| BAT | Both | GWAS Catalog | 382,988 | EUR | 32042192 | 120 |
| BAT | Male | GWAS Catalog | 178,782 | EUR | 32042192 | 93 |
| BAT | Female | GWAS Catalog | 188,507 | EUR | 32042192 | 147 |
| TT | Both | GWAS Catalog | 425,097 | EUR | 32042192 | 179 |
| TT | Male | GWAS Catalog | 194,453 | EUR | 32042192 | 182 |
| TT | Female | GWAS Catalog | 230,454 | EUR | 32042192 | 216 |
| AMH | Female | GWAS Catalog | 7,049 | EUR | 35274129 | 4 |
| ER | Both | GWAS Catalog | 3,301 | EUR | 29875488 | 9 |
| PRLR | Both | IEU | 3,301 | EUR | 29875488 | 8 |
| SHBG | Both | GWAS Catalog | 370,125 | EUR | 32042192 | 403 |
| SHBG | Male | GWAS Catalog | 180,726 | EUR | 32042192 | 248 |
| SHBG | Female | GWAS Catalog | 189,473 | EUR | 32042192 | 226 |
| IBD | Both | IEU | 12,882/21,770 | EUR | 26192919 | 63 |
| CD | Both | IEU | 5,956/14,927 | EUR | 26192919 | 53 |
| UC | Both | IEU | 6,968/20,464 | EUR | 26192919 | 38 |

Abbreviations: FSH, follicle-stimulating hormone; LH, luteinizing hormone; E2, estradiol; PROG, progesterone; PRL, prolactin; BAT, bioavailable testosterone; TT, total testosterone; AMH, anti-Müllerian hormone; ER, estrogen receptor; PRLR, prolactin receptor; SHBG, sex-hormone binding globulin; IBD, inflammatory bowel disease; CD, crohn’s disease; UC, ulcerative colitis; IEU, integrative epidemiology unit; GWAS, genome-wide association study; EUR, European; PMID, PubMed unique identifier; IVs, instrumental variables.

**Table S2. Heterogeneity and pleiotropy tests for the associations between sex hormones and IBD/CD/UC.**

| Exposure | Outcome | Cochran’s Q test | | MR PRESSO test | | MR Egger test | |
| --- | --- | --- | --- | --- | --- | --- | --- |
|  |  | Q | *P* value | Global test  *P* value | Distortion test  *P* value | Intercept | *P* value |
| FSH | IBD | 0.377 | 0.539 | - | - | - | - |
|  | CD | 0.154 | 0.694 | - | - | - | - |
|  | UC | 0.495 | 0.482 | - | - | - | - |
| LH | IBD | 8.334 | 0.304 | 0.398 | - | -0.000 | 0.985 |
|  | CD | 5.894 | 0.552 | 0.562 | - | -0.017 | 0.554 |
|  | UC | 9.221 | 0.237 | 0.339 | - | 0.030 | 0.318 |
| E2 | IBD | 10.920 | 0.536 | 0.597 | - | -0.005 | 0.833 |
|  | CD | 6.202 | 0.906 | 0.911 | - | -0.007 | 0.809 |
|  | UC | 10.735 | 0.552 | 0.599 | - | -0.022 | 0.434 |
| PROG | IBD | 0.584 | 0.445 | - | - | - | - |
|  | CD | 1.242 | 0.265 | - | - | - | - |
|  | UC | 0.491 | 0.483 | - | - | - | - |
| PRL | IBD | 38.487 | <0.001 | <0.001 | 0.201 | -0.026 | 0.331 |
|  | CD | 24.431 | 0.041 | 0.045 | - | -0.011 | 0.701 |
|  | UC | 27.070 | 0.019 | 0.028 | 0.132 | -0.022 | 0.432 |
| BAT | IBD | 186.225 | <0.001 | <0.001 | 0.888 | 0.006 | 0.280 |
|  | CD | 147.211 | 0.004 | 0.004 | 0.874 | 0.007 | 0.309 |
|  | UC | 169.808 | <0.001 | <0.001 | 0.078 | 0.004 | 0.568 |
| TT | IBD | 331.662 | <0.001 | <0.001 | 0.351 | 0.009 | 0.088 |
|  | CD | 296.640 | <0.001 | <0.001 | 0.928 | 0.006 | 0.369 |
|  | UC | 280.887 | <0.001 | <0.001 | 0.746 | 0.011 | 0.052 |

Abbreviations: FSH, follicle-stimulating hormone; LH, luteinizing hormone; E2, estradiol; PROG, progesterone; PRL, prolactin; BAT, bioavailable testosterone; TT, total testosterone; IBD, inflammatory bowel disease; CD, crohn’s disease; UC, ulcerative colitis.

**Table S3. Causal effects of sex hormones on IBD/CD/UC using different models.**

| Exposure | Outcome | n_SNPs | Model | OR | OR 95% CI | *P* value |
| --- | --- | --- | --- | --- | --- | --- |
| FSH | IBD | 2 | IVW | 0.994 | 0.835, 1.183 | 0.946 |
|  | CD | 2 | IVW | 0.846 | 0.665, 1.077 | 0.175 |
|  | UC | 2 | IVW | 1.069 | 0.861, 1.328 | 0.546 |
| LH | IBD | 8 | IVW | 1.042 | 0.961, 1.130 | 0.321 |
|  |  |  | MR Egger | 1.043 | 0.895, 1.216 | 0.609 |
|  |  |  | Weighted median | 1.066 | 0.975, 1.166 | 0.160 |
|  | CD | 8 | IVW | 1.065 | 0.960, 1.181 | 0.234 |
|  |  |  | MR Egger | 1.118 | 0.931, 1.342 | 0.279 |
|  |  |  | Weighted median | 1.120 | 0.989, 1.269 | 0.074 |
|  | UC | 8 | IVW | 1.033 | 0.929, 1.147 | 0.551 |
|  |  |  | MR Egger | 0.950 | 0.792, 1.140 | 0.603 |
|  |  |  | Weighted median | 1.012 | 0.907, 1.129 | 0.829 |
| E2 | IBD | 13 | IVW | 0.802 | 0.527, 1.218 | 0.300 |
|  |  |  | MR Egger | 0.906 | 0.275, 2.983 | 0.874 |
|  |  |  | Weighted median | 0.741 | 0.426, 1.288 | 0.288 |
|  | CD | 13 | IVW | 0.531 | 0.302, 0.933 | **0.028*** |
|  |  |  | MR Egger | 0.641 | 0.129, 3.174 | 0.597 |
|  |  |  | Weighted median | 0.649 | 0.307, 1.369 | 0.256 |
|  | UC | 13 | IVW | 1.120 | 0.660, 1.901 | 0.675 |
|  |  |  | MR Egger | 2.008 | 0.446, 9.042 | 0.383 |
|  |  |  | Weighted median | 1.249 | 0.626, 2.491 | 0.528 |
| PROG | IBD | 2 | IVW | 1.011 | 0.793, 1.288 | 0.933 |
|  | CD | 2 | IVW | 1.019 | 0.700, 1.484 | 0.920 |
|  | UC | 2 | IVW | 1.010 | 0.747, 1.366 | 0.948 |
| PRL | IBD | 15 | IVW | 0.934 | 0.784, 1.114 | 0.448 |
|  |  |  | MR Egger | 1.107 | 0.762, 1.609 | 0.602 |
|  |  |  | Weighted median | 0.991 | 0.842, 1.165 | 0.910 |
|  | CD | 15 | IVW | 0.917 | 0.758, 1.108 | 0.369 |
|  |  |  | MR Egger | 0.987 | 0.650, 1.499 | 0.952 |
|  |  |  | Weighted median | 1.023 | 0.829, 1.262 | 0.834 |
|  | UC | 15 | IVW | 0.961 | 0.798, 1.157 | 0.674 |
|  |  |  | MR Egger | 1.110 | 0.747, 1.650 | 0.614 |
|  |  |  | Weighted median | 0.993 | 0.825, 1.197 | 0.945 |
| BAT | IBD | 106 | IVW | 0.971 | 0.751, 1.254 | 0.820 |
|  |  |  | MR Egger | 0.745 | 0.434, 1.281 | 0.290 |
|  |  |  | Weighted median | 1.104 | 0.772, 1.579 | 0.588 |
|  | CD | 106 | IVW | 0.891 | 0.653, 1.215 | 0.465 |
|  |  |  | MR Egger | 0.657 | 0.339, 1.272 | 0.215 |
|  |  |  | Weighted median | 1.015 | 0.655, 1.574 | 0.946 |
|  | UC | 107 | IVW | 0.918 | 0.676, 1.246 | 0.583 |
|  |  |  | MR Egger | 0.777 | 0.407, 1.484 | 0.446 |
|  |  |  | Weighted median | 0.944 | 0.595, 1.495 | 0.804 |
| TT | IBD | 161 | IVW | 0.774 | 0.548, 1.093 | 0.146 |
|  |  |  | MR Egger | 0.435 | 0.207, 0.913 | **0.029*** |
|  |  |  | Weighted median | 0.702 | 0.443, 1.114 | 0.133 |
|  | CD | 161 | IVW | 0.618 | 0.396, 0.965 | **0.034*** |
|  |  |  | MR Egger | 0.417 | 0.159, 1.095 | 0.078 |
|  |  |  | Weighted median | 0.683 | 0.393, 1.185 | 0.175 |
|  | UC | 161 | IVW | 0.876 | 0.588, 1.305 | 0.515 |
|  |  |  | MR Egger | 0.411 | 0.175, 0.966 | **0.043*** |
|  |  |  | Weighted median | 0.814 | 0.492, 1.347 | 0.422 |

**P*<0.05. Abbreviations: FSH, follicle-stimulating hormone; LH, luteinizing hormone; E2, estradiol; PROG, progesterone; PRL, prolactin; BAT, bioavailable testosterone; TT, total testosterone; IBD, inflammatory bowel disease; CD, crohn’s disease; UC, ulcerative colitis; OR, odds ratio; CI, confidence interval; IVW, inverse variance weighted.

**Table S4. Causal effects of ER, PRLR and SHBG on IBD/CD/UC using different models.**

| Exposure | Outcome | n_SNPs | Model | OR | OR 95% CI | *P* value |
| --- | --- | --- | --- | --- | --- | --- |
| ER | IBD | 8 | IVW | 1.076 | 0.921, 1.258 | 0.355 |
|  |  |  | MR Egger | 0.806 | 0.399, 1.628 | 0.570 |
|  |  |  | Weighted median | 1.110 | 0.945, 1.303 | 0.203 |
|  | CD | 8 | IVW | 1.055 | 0.803, 1.386 | 0.700 |
|  |  |  | MR Egger | 0.473 | 0.151, 1.487 | 0.248 |
|  |  |  | Weighted median | 0.985 | 0.785, 1.235 | 0.895 |
|  | UC | 8 | IVW | 1.094 | 0.953, 1.254 | 0.201 |
|  |  |  | MR Egger | 1.084 | 0.568, 2.067 | 0.815 |
|  |  |  | Weighted median | 1.071 | 0.885, 1.296 | 0.482 |
| PRLR | IBD | 7 | IVW | 1.013 | 0.902, 1.138 | 0.830 |
|  |  |  | MR Egger | 1.002 | 0.741, 1.355 | 0.992 |
|  |  |  | Weighted median | 1.012 | 0.874, 1.171 | 0.878 |
|  | CD | 7 | IVW | 1.067 | 0.921, 1.235 | 0.388 |
|  |  |  | MR Egger | 0.891 | 0.626, 1.269 | 0.551 |
|  |  |  | Weighted median | 1.120 | 0.920, 1.363 | 0.258 |
|  | UC | 7 | IVW | 1.041 | 0.900, 1.204 | 0.587 |
|  |  |  | MR Egger | 1.139 | 0.791, 1.638 | 0.515 |
|  |  |  | Weighted median | 1.038 | 0.857, 1.256 | 0.705 |
| SHBG | IBD | 368 | IVW | 0.919 | 0.732, 1.153 | 0.464 |
|  |  |  | MR Egger | 0.913 | 0.624, 1.337 | 0.641 |
|  |  |  | Weighted median | 0.891 | 0.653, 1.215 | 0.465 |
|  | CD | 369 | IVW | 0.754 | 0.559, 1.015 | 0.063 |
|  |  |  | MR Egger | 0.674 | 0.409, 1.112 | 0.123 |
|  |  |  | Weighted median | 0.878 | 0.574, 1.345 | 0.550 |
|  | UC | 370 | IVW | 1.074 | 0.818, 1.410 | 0.608 |
|  |  |  | MR Egger | 1.093 | 0.691, 1.729 | 0.703 |
|  |  |  | Weighted median | 0.944 | 0.636, 1.403 | 0.777 |

Abbreviations: ER, estrogen receptor; PRLR, prolactin receptor; SHBG, sex hormone-binding globulin; IBD, inflammatory bowel disease; CD, Crohn’s disease; UC, ulcerative colitis; OR, odds ratio; CI, confidence interval; IVW, inverse variance weighted.

**Table S5. Heterogeneity and pleiotropy tests for the associations between ER/PRLR/SHBG and IBD/CD/UC.**

| Exposure | Outcome | Cochran’s Q test | | MR PRESSO test | | MR Egger test | |
| --- | --- | --- | --- | --- | --- | --- | --- |
|  |  | Q | *P* value | Global test  *P* value | Distortion test  *P* value | Intercept | *P* value |
| ER | IBD | 14.370 | 0.045 | 0.066 | - | 0.044 | 0.440 |
|  | CD | 23.208 | 0.002 | 0.002 | 0.126 | 0.120 | 0.209 |
|  | UC | 7.098 | 0.419 | 0.403 | - | 0.001 | 0.979 |
| PRLR | IBD | 7.122 | 0.310 | 0.270 | - | 0.002 | 0.939 |
|  | CD | 5.258 | 0.511 | 0.484 | - | 0.035 | 0.323 |
|  | UC | 7.117 | 0.310 | 0.312 | - | -0.018 | 0.617 |
| SHBG | IBD | 624.208 | <0.001 | <0.001 | 0.017 | 0.000 | 0.970 |
|  | CD | 583.273 | <0.001 | <0.001 | 0.901 | 0.002 | 0.588 |
|  | UC | 573.738 | <0.001 | <0.001 | 0.694 | -0.000 | 0.925 |

Abbreviations: ER, estrogen receptor; PRLR, prolactin receptor; SHBG, sex hormone-binding globulin; IBD, inflammatory bowel disease; CD, Crohn’s disease; UC, ulcerative colitis.

**Table S6. Heterogeneity and pleiotropy tests for the sex-stratified associations between E2/PROG/BAT/TT/SHBG/AMH and IBD/CD/UC.**

| Exposure | Exp_sex | Outcome | Cochran’s Q test | | MR PRESSO test | | MR Egger test | |
| --- | --- | --- | --- | --- | --- | --- | --- | --- |
|  |  |  | Q | *P* value | Global test  *P* value | Distortion test  *P* value | Intercept | *P* value |
| E2 | Male | IBD | 15.709 | 0.152 | 0.191 | - | -0.013 | 0.615 |
|  | Female |  | 10.551 | 0.001 | - | - | - | - |
|  | Male | CD | 17.380 | 0.097 | 0.085 | - | -0.023 | 0.529 |
|  | Female |  | 4.916 | 0.027 | - | - | - | - |
|  | Male | UC | 16.586 | 0.121 | 0.146 | - | 0.013 | 0.694 |
|  | Female |  | 9.888 | 0.002 | - | - | - | - |
| PROG | Male | IBD | 11.753 | 0.109 | 0.128 | - | -0.003 | 0.920 |
|  | Female |  | 3.257 | 0.196 | - | - | -0.115 | 0.343 |
|  | Male | CD | 12.911 | 0.074 | 0.081 | - | -0.026 | 0.598 |
|  | Female |  | 6.818 | 0.033 | - | - | -0.226 | 0.243 |
|  | Male | UC | 8.691 | 0.276 | 0.286 | - | 0.013 | 0.722 |
|  | Female |  | 0.481 | 0.786 | - | - | -0.059 | 0.619 |
| BAT | Male | IBD | 134.909 | <0.001 | <0.001 | 0.083 | -0.009 | 0.122 |
|  | Female |  | 231.920 | <0.001 | <0.001 | 0.856 | 0.002 | 0.725 |
|  | Male | CD | 132.961 | <0.001 | <0.001 | 0.764 | -0.003 | 0.752 |
|  | Female |  | 193.752 | <0.001 | <0.001 | - | 0.000 | 0.953 |
|  | Male | UC | 110.635 | 0.013 | 0.014 | 0.149 | -0.012 | 0.056 |
|  | Female |  | 193.176 | <0.001 | <0.001 | - | 0.003 | 0.653 |
| TT | Male | IBD | 313.037 | <0.001 | <0.001 | 0.966 | 0.003 | 0.468 |
|  | Female |  | 384.881 | <0.001 | <0.001 | 0.663 | -0.001 | 0.887 |
|  | Male | CD | 271.578 | <0.001 | <0.001 | 0.886 | 0.004 | 0.420 |
|  | Female |  | 358.538 | <0.001 | <0.001 | 0.488 | -0.006 | 0.274 |
|  | Male | UC | 277.926 | <0.001 | <0.001 | 0.985 | 0.004 | 0.490 |
|  | Female |  | 327.501 | <0.001 | <0.001 | 0.840 | 0.005 | 0.308 |
| SHBG | Male | IBD | 421.344 | <0.001 | <0.001 | 0.876 | 0.002 | 0.573 |
|  | Female |  | 365.574 | <0.001 | <0.001 | 0.261 | 0.005 | 0.125 |
|  | Male | CD | 387.126 | <0.001 | <0.001 | 0.980 | 0.003 | 0.530 |
|  | Female |  | 357.073 | <0.001 | <0.001 | 0.960 | 0.008 | 0.078 |
|  | Male | UC | 369.307 | <0.001 | <0.001 | 0.882 | 0.001 | 0.795 |
|  | Female |  | 333.590 | <0.001 | <0.001 | 0.393 | 0.004 | 0.388 |
| AMH | Female | IBD | 8.680 | 0.034 | 0.081 | - | -0.059 | 0.267 |
|  | Female | CD | 3.229 | 0.358 | 0.441 | - | -0.050 | 0.314 |
|  | Female | UC | 7.833 | 0.050 | 0.102 | - | -0.068 | 0.296 |

Abbreviations: E2, estradiol; PROG, progesterone; BAT, bioavailable testosterone; TT, total testosterone; SHBG, sex hormone-binding globulin; AMH, anti-Müllerian hormone; IBD, inflammatory bowel disease; CD, Crohn’s disease; UC, ulcerative colitis.

**Table S7. Sex-stratified causal effects of E2 on IBD/CD/UC using different models.**

| Exposure | Exp_sex | Outcome | n_SNPs | Model | OR | OR 95% CI | *P* value |
| --- | --- | --- | --- | --- | --- | --- | --- |
| E2 | Male | IBD | 12 | IVW | 0.890 | 0.798, 0.994 | **0.038*** |
|  |  |  |  | MR Egger | 0.966 | 0.695, 1.343 | 0.843 |
|  |  |  |  | Weighted median | 0.936 | 0.826, 1.062 | 0.307 |
|  | Female |  | 2 | IVW | 0.843 | 0.284, 2.504 | 0.758 |
|  | Male | CD | 12 | IVW | 0.830 | 0.709, 0.972 | **0.020*** |
|  |  |  |  | MR Egger | 0.962 | 0.600, 1.542 | 0.874 |
|  |  |  |  | Weighted median | 0.966 | 0.815, 1.145 | 0.687 |
|  | Female |  | 2 | IVW | 0.773 | 0.281, 2.121 | 0.617 |
|  | Male | UC | 12 | IVW | 0.945 | 0.820, 1.089 | 0.436 |
|  |  |  |  | MR Egger | 0.870 | 0.568, 1.333 | 0.538 |
|  |  |  |  | Weighted median | 0.911 | 0.778, 1.067 | 0.246 |
|  | Female |  | 2 | IVW | 0.861 | 0.229, 3.231 | 0.824 |

**P*<0.05. Abbreviations: E2, estradiol; IBD, inflammatory bowel disease; CD, Crohn’s disease; UC, ulcerative colitis; OR, odds ratio; CI, confidence interval; IVW, inverse variance weighted.

**Table S8. Sex-stratified causal effects of PROG on IBD/CD/UC using different models.**

| Exposure | Exp_sex | Outcome | n_SNPs | Model | OR | OR 95% CI | *P* value |
| --- | --- | --- | --- | --- | --- | --- | --- |
| PROG | Male | IBD | 8 | IVW | 1.047 | 0.860, 1.275 | 0.645 |
|  |  |  |  | MR Egger | 1.077 | 0.615, 1.884 | 0.804 |
|  |  |  |  | Weighted median | 1.021 | 0.828, 1.259 | 0.845 |
|  | Female |  | 3 | IVW | 1.030 | 0.881, 1.203 | 0.712 |
|  |  |  |  | MR Egger | 1.325 | 0.963, 1.824 | 0.334 |
|  |  |  |  | Weighted median | 1.033 | 0.891, 1.197 | 0.672 |
|  | Male | CD | 8 | IVW | 1.069 | 0.805, 1.419 | 0.647 |
|  |  |  |  | MR Egger | 1.320 | 0.593, 2.939 | 0.522 |
|  |  |  |  | Weighted median | 0.873 | 0.656, 1.163 | 0.353 |
|  | Female |  | 3 | IVW | 1.147 | 0.851, 1.546 | 0.367 |
|  |  |  |  | MR Egger | 1.873 | 1.234, 2.845 | 0.208 |
|  |  |  |  | Weighted median | 1.158 | 0.949, 1.413 | 0.148 |
|  | Male | UC | 8 | IVW | 0.994 | 0.804, 1.229 | 0.958 |
|  |  |  |  | MR Egger | 0.896 | 0.495, 1.621 | 0.729 |
|  |  |  |  | Weighted median | 0.968 | 0.747, 1.255 | 0.808 |
|  | Female |  | 3 | IVW | 0.958 | 0.820, 1.119 | 0.587 |
|  |  |  |  | MR Egger | 1.093 | 0.726, 1.646 | 0.744 |
|  |  |  |  | Weighted median | 0.971 | 0.812, 1.161 | 0.744 |

Abbreviations: PROG, progesterone; IBD, inflammatory bowel disease; CD, Crohn’s disease; UC, ulcerative colitis; OR, odds ratio; CI, confidence interval; IVW, inverse variance weighted.

**Table S9. Sex-stratified causal effects of BAT on IBD/CD/UC using different models.**

| Exposure | Exp_sex | Outcome | n_SNPs | Model | OR | OR 95% CI | *P* value |
| --- | --- | --- | --- | --- | --- | --- | --- |
| BAT | Male | IBD | 80 | IVW | 1.017 | 0.856, 1.209 | 0.848 |
|  |  |  |  | MR Egger | 1.267 | 0.916, 1.752 | 0.157 |
|  |  |  |  | Weighted median | 0.893 | 0.711, 1.121 | 0.329 |
|  | Female |  | 130 | IVW | 0.821 | 0.685, 0.985 | **0.034*** |
|  |  |  |  | MR Egger | 0.775 | 0.537, 1.119 | 0.177 |
|  |  |  |  | Weighted median | 0.916 | 0.706, 1.188 | 0.508 |
|  | Male | CD | 80 | IVW | 0.970 | 0.766, 1.228 | 0.798 |
|  |  |  |  | MR Egger | 1.033 | 0.655, 1.629 | 0.890 |
|  |  |  |  | Weighted median | 0.899 | 0.667, 1.211 | 0.483 |
|  | Female |  | 130 | IVW | 0.795 | 0.634, 0.997 | **0.047*** |
|  |  |  |  | MR Egger | 0.785 | 0.497, 1.242 | 0.304 |
|  |  |  |  | Weighted median | 0.840 | 0.600, 1.177 | 0.311 |
|  | Male | UC | 81 | IVW | 0.997 | 0.821, 1.210 | 0.975 |
|  |  |  |  | MR Egger | 1.348 | 0.941, 1.930 | 0.108 |
|  |  |  |  | Weighted median | 1.029 | 0.775, 1.367 | 0.842 |
|  | Female |  | 131 | IVW | 0.802 | 0.652, 0.986 | **0.036*** |
|  |  |  |  | MR Egger | 0.738 | 0.486, 1.120 | 0.156 |
|  |  |  |  | Weighted median | 0.870 | 0.616, 1.227 | 0.426 |

**P*<0.05. Abbreviations: BAT, bioavailable testosterone; IBD, inflammatory bowel disease; CD, Crohn’s disease; UC, ulcerative colitis; OR, odds ratio; CI, confidence interval; IVW, inverse variance weighted.

**Table S10. Sex-stratified causal effects of TT on IBD/CD/UC using different models.**

| Exposure | Exp_sex | Outcome | n_SNPs | Model | OR | OR 95% CI | *P* value |
| --- | --- | --- | --- | --- | --- | --- | --- |
| TT | Male | IBD | 160 | IVW | 1.015 | 0.886, 1.164 | 0.826 |
|  |  |  |  | MR Egger | 0.936 | 0.723, 1.212 | 0.618 |
|  |  |  |  | Weighted median | 0.922 | 0.774, 1.099 | 0.367 |
|  | Female |  | 200 | IVW | 0.865 | 0.762, 0.982 | **0.025*** |
|  |  |  |  | MR Egger | 0.878 | 0.689, 1.119 | 0.294 |
|  |  |  |  | Weighted median | 0.933 | 0.786, 1.107 | 0.424 |
|  | Male | CD | 160 | IVW | 0.934 | 0.784, 1.112 | 0.442 |
|  |  |  |  | MR Egger | 0.831 | 0.596, 1.159 | 0.277 |
|  |  |  |  | Weighted median | 0.992 | 0.784, 1.255 | 0.945 |
|  | Female |  | 200 | IVW | 0.831 | 0.703, 0.982 | **0.029*** |
|  |  |  |  | MR Egger | 0.967 | 0.703, 1.329 | 0.835 |
|  |  |  |  | Weighted median | 0.914 | 0.728, 1.147 | 0.437 |
|  | Male | UC | 161 | IVW | 1.058 | 0.901, 1.242 | 0.494 |
|  |  |  |  | MR Egger | 0.966 | 0.714, 1.308 | 0.824 |
|  |  |  |  | Weighted median | 1.023 | 0.817, 1.279 | 0.845 |
|  | Female |  | 200 | IVW | 0.874 | 0.754, 1.012 | 0.073 |
|  |  |  |  | MR Egger | 0.772 | 0.583, 1.021 | 0.071 |
|  |  |  |  | Weighted median | 0.852 | 0.689, 1.053 | 0.139 |

**P*<0.05. Abbreviations: TT, total testosterone; IBD, inflammatory bowel disease; CD, Crohn’s disease; UC, ulcerative colitis; OR, odds ratio; CI, confidence interval; IVW, inverse variance weighted.

**Table S11. Sex-stratified causal effects of SHBG on IBD/CD/UC using different models.**

| Exposure | Exp_sex | Outcome | n_SNPs | Model | OR | OR 95% CI | *P* value |
| --- | --- | --- | --- | --- | --- | --- | --- |
| SHBG | Male | IBD | 224 | IVW | 0.880 | 0.692, 1.120 | 0.300 |
|  |  |  |  | MR Egger | 0.800 | 0.530, 1.207 | 0.289 |
|  |  |  |  | Weighted median | 0.790 | 0.580, 1.078 | 0.137 |
|  | Female |  | 203 | IVW | 0.976 | 0.772, 1.235 | 0.842 |
|  |  |  |  | MR Egger | 0.741 | 0.486, 1.130 | 0.165 |
|  |  |  |  | Weighted median | 1.052 | 0.766, 1.443 | 0.755 |
|  | Male | CD | 225 | IVW | 0.692 | 0.506, 0.946 | **0.021*** |
|  |  |  |  | MR Egger | 0.602 | 0.352, 1.028 | 0.064 |
|  |  |  |  | Weighted median | 0.527 | 0.346, 0.805 | **0.003*** |
|  | Female |  | 203 | IVW | 0.841 | 0.613, 1.153 | 0.282 |
|  |  |  |  | MR Egger | 0.550 | 0.313, 0.968 | **0.039*** |
|  |  |  |  | Weighted median | 0.857 | 0.545, 1.349 | 0.505 |
|  | Male | UC | 226 | IVW | 1.005 | 0.758, 1.332 | 0.974 |
|  |  |  |  | MR Egger | 0.954 | 0.589, 1.546 | 0.848 |
|  |  |  |  | Weighted median | 1.017 | 0.680, 1.522 | 0.933 |
|  | Female |  | 206 | IVW | 1.078 | 0.815, 1.425 | 0.599 |
|  |  |  |  | MR Egger | 0.895 | 0.540, 1.484 | 0.668 |
|  |  |  |  | Weighted median | 0.965 | 0.643, 1.448 | 0.863 |

**P*<0.05. Abbreviations: SHBG, sex hormone-binding globulin; IBD, inflammatory bowel disease; CD, Crohn’s disease; UC, ulcerative colitis; OR, odds ratio; CI, confidence interval; IVW, inverse variance weighted.

**Table S12. Causal effects of AMH in female on IBD/CD/UC using different models.**

| Exposure | Exp_sex | Outcome | n_SNPs | Model | OR | OR 95% CI | *P* value |
| --- | --- | --- | --- | --- | --- | --- | --- |
| AMH | Female | IBD | 4 | IVW | 0.937 | 0.658, 1.334 | 0.718 |
|  |  |  |  | MR Egger | 1.514 | 0.764, 3.002 | 0.357 |
|  |  |  |  | Weighted median | 0.905 | 0.694, 1.178 | 0.457 |
|  | Female | CD | 4 | IVW | 0.958 | 0.714, 1.285 | 0.773 |
|  |  |  |  | MR Egger | 1.432 | 0.743, 2.761 | 0.396 |
|  |  |  |  | Weighted median | 0.977 | 0.695, 1.374 | 0.895 |
|  | Female | UC | 4 | IVW | 0.956 | 0.627, 1.458 | 0.835 |
|  |  |  |  | MR Egger | 1.664 | 0.706, 3.918 | 0.364 |
|  |  |  |  | Weighted median | 0.857 | 0.618, 1.188 | 0.354 |

Abbreviations: AMH, anti-Müllerian hormone; IBD, inflammatory bowel disease; CD, Crohn’s disease; UC, ulcerative colitis; OR, odds ratio; CI, confidence interval; IVW, inverse variance weighted.

**Table S13. Causal effects of IBD/CD/UC on sex hormones using different models.**

| Exposure | Outcome | n_SNPs | Model | Beta | 95% CI | *P* value |
| --- | --- | --- | --- | --- | --- | --- |
| IBD | FSH | 63 | IVW | -0.031 | -0.081, 0.019 | 0.231 |
|  |  |  | MR Egger | -0.111 | -0.237, 0.015 | 0.089 |
|  |  |  | Weighted median | -0.022 | -0.094, 0.050 | 0.545 |
| CD |  | 52 | IVW | -0.024 | -0.063, 0.015 | 0.235 |
|  |  |  | MR Egger | -0.022 | -0.112, 0.068 | 0.639 |
|  |  |  | Weighted median | -0.004 | -0.064, 0.056 | 0.892 |
| UC |  | 37 | IVW | -0.031 | -0.081, 0.020 | 0.237 |
|  |  |  | MR Egger | -0.078 | -0.201, 0.044 | 0.219 |
|  |  |  | Weighted median | -0.026 | -0.095, 0.044 | 0.468 |
| IBD | LH | 63 | IVW | -0.015 | -0.062, 0.032 | 0.522 |
|  |  |  | MR Egger | -0.077 | -0.196, 0.041 | 0.206 |
|  |  |  | Weighted median | -0.013 | -0.082, 0.056 | 0.713 |
| CD |  | 52 | IVW | -0.013 | -0.057, 0.031 | 0.556 |
|  |  |  | MR Egger | -0.019 | -0.121, 0.082 | 0.711 |
|  |  |  | Weighted median | -0.007 | -0.065, 0.050 | 0.807 |
| UC |  | 37 | IVW | -0.021 | -0.070, 0.028 | 0.400 |
|  |  |  | MR Egger | -0.061 | -0.180, 0.059 | 0.328 |
|  |  |  | Weighted median | -0.028 | -0.100, 0.043 | 0.434 |
| IBD | E2 | 63 | IVW | 0.001 | -0.008, 0.010 | 0.834 |
|  |  |  | MR Egger | 0.017 | -0.007, 0.041 | 0.167 |
|  |  |  | Weighted median | 0.001 | -0.012, 0.015 | 0.833 |
| CD |  | 52 | IVW | 0.002 | -0.007, 0.010 | 0.704 |
|  |  |  | MR Egger | 0.011 | -0.009, 0.030 | 0.291 |
|  |  |  | Weighted median | 0.005 | -0.006, 0.016 | 0.400 |
| UC |  | 37 | IVW | -0.004 | -0.013, 0.005 | 0.390 |
|  |  |  | MR Egger | 0.008 | -0.013, 0.030 | 0.443 |
|  |  |  | Weighted median | -0.007 | -0.020, 0.006 | 0.274 |
| IBD | PROG | 55 | IVW | -0.019 | -0.053, 0.015 | 0.278 |
|  |  |  | MR Egger | -0.062 | -0.157, 0.032 | 0.202 |
|  |  |  | Weighted median | -0.017 | -0.068, 0.034 | 0.515 |
| CD |  | 47 | IVW | -0.001 | -0.031, 0.028 | 0.937 |
|  |  |  | MR Egger | -0.014 | -0.081, 0.054 | 0.695 |
|  |  |  | Weighted median | 0.003 | -0.038, 0.043 | 0.897 |
| UC |  | 30 | IVW | -0.031 | -0.069, 0.006 | 0.099 |
|  |  |  | MR Egger | -0.050 | -0.154, 0.055 | 0.360 |
|  |  |  | Weighted median | -0.052 | -0.106, 0.002 | 0.058 |
| IBD | PRL | 63 | IVW | -0.012 | -0.041, 0.018 | 0.435 |
|  |  |  | MR Egger | 0.025 | -0.058, 0.109 | 0.552 |
|  |  |  | Weighted median | 0.006 | -0.035, 0.047 | 0.791 |
| CD |  | 52 | IVW | -0.007 | -0.033, 0.019 | 0.594 |
|  |  |  | MR Egger | 0.014 | -0.047, 0.076 | 0.649 |
|  |  |  | Weighted median | 0.011 | -0.023, 0.046 | 0.527 |
| UC |  | 37 | IVW | -0.014 | -0.050, 0.022 | 0.441 |
|  |  |  | MR Egger | 0.015 | -0.088, 0.118 | 0.782 |
|  |  |  | Weighted median | -0.012 | -0.056, 0.031 | 0.579 |
| IBD | BAT | 63 | IVW | -0.001 | -0.007, 0.005 | 0.712 |
|  |  |  | MR Egger | 0.007 | -0.008, 0.023 | 0.353 |
|  |  |  | Weighted median | 0.005 | -0.001, 0.011 | 0.117 |
| CD |  | 52 | IVW | -0.004 | -0.009, 0.001 | 0.158 |
|  |  |  | MR Egger | 0.006 | -0.006, 0.018 | 0.317 |
|  |  |  | Weighted median | 0.001 | -0.004, 0.006 | 0.775 |
| UC |  | 37 | IVW | -0.002 | -0.008, 0.005 | 0.597 |
|  |  |  | MR Egger | 0.000 | -0.015, 0.016 | 0.961 |
|  |  |  | Weighted median | 0.009 | 0.002, 0.016 | **0.012*** |
| IBD | TT | 63 | IVW | -0.000 | -0.004, 0.003 | 0.857 |
|  |  |  | MR Egger | 0.003 | -0.007, 0.012 | 0.581 |
|  |  |  | Weighted median | -0.000 | -0.004, 0.004 | 0.885 |
| CD |  | 52 | IVW | -0.002 | -0.006, 0.002 | 0.296 |
|  |  |  | MR Egger | -0.003 | -0.012, 0.007 | 0.589 |
|  |  |  | Weighted median | -0.002 | -0.005, 0.001 | 0.162 |
| UC |  | 37 | IVW | 0.001 | -0.003, 0.005 | 0.709 |
|  |  |  | MR Egger | 0.005 | -0.004, 0.014 | 0.311 |
|  |  |  | Weighted median | -0.000 | -0.004, 0.004 | 0.979 |

**P*<0.05. Abbreviations: IBD, inflammatory bowel disease; CD, Crohn’s disease; UC, ulcerative colitis; FSH, follicle-stimulating hormone; LH, luteinizing hormone; E2, estradiol; PROG, progesterone; PRL, prolactin; BAT, bioavailable testosterone; TT, total testosterone; CI, confidence interval; IVW, inverse variance weighted.

**Table S14. Heterogeneity and pleiotropy tests for the associations between IBD/CD/UC and sex hormones.**

| Exposure | Outcome | Cochran’s Q test | | MR PRESSO test | | MR Egger test | |
| --- | --- | --- | --- | --- | --- | --- | --- |
|  |  | Q | *P* value | Global test  *P* value | Distortion test  *P* value | Intercept | *P* value |
| IBD | FSH | 79.505 | 0.066 | 0.055 | - | 0.014 | 0.178 |
| CD |  | 54.865 | 0.330 | 0.340 | - | -0.000 | 0.962 |
| UC |  | 42.712 | 0.205 | 0.219 | - | 0.010 | 0.408 |
| IBD | LH | 69.634 | 0.236 | 0.254 | - | 0.011 | 0.270 |
| CD |  | 70.286 | 0.038 | 0.034 | 0.079 | 0.001 | 0.897 |
| UC |  | 40.187 | 0.290 | 0.279 | - | 0.008 | 0.483 |
| IBD | E2 | 84.407 | 0.031 | 0.032 | - | -0.003 | 0.157 |
| CD |  | 78.854 | 0.007 | 0.009 | - | -0.002 | 0.321 |
| UC |  | 40.047 | 0.295 | 0.304 | - | -0.003 | 0.220 |
| IBD | PROG | 57.085 | 0.361 | 0.379 | - | 0.007 | 0.338 |
| CD |  | 54.335 | 0.187 | 0.155 | - | 0.003 | 0.690 |
| UC |  | 30.884 | 0.371 | 0.383 | - | 0.004 | 0.717 |
| IBD | PRL | 73.138 | 0.157 | 0.148 | - | -0.006 | 0.354 |
| CD |  | 64.503 | 0.097 | 0.094 | - | -0.005 | 0.457 |
| UC |  | 52.932 | 0.034 | 0.038 | 0.125 | -0.006 | 0.565 |
| IBD | BAT | 180.217 | <0.001 | <0.001 | 0.072 | -0.001 | 0.243 |
| CD |  | 155.522 | <0.001 | <0.001 | 0.969 | -0.002 | 0.073 |
| UC |  | 108.343 | <0.001 | <0.001 | 0.226 | -0.000 | 0.771 |
| IBD | TT | 169.026 | <0.001 | <0.001 | 0.787 | -0.001 | 0.497 |
| CD |  | 205.055 | <0.001 | <0.001 | 0.828 | 0.000 | 0.922 |
| UC |  | 95.063 | <0.001 | <0.001 | 0.079 | -0.001 | 0.344 |

Abbreviations: IBD, inflammatory bowel disease; CD, Crohn’s disease; UC, ulcerative colitis; FSH, follicle-stimulating hormone; LH, luteinizing hormone; E2, estradiol; PROG, progesterone; PRL, prolactin; BAT, bioavailable testosterone; TT, total testosterone.

**Table S15. Causal effects of IBD/CD/UC on ER, PRLR and SHBG using different models.**

| Exposure | Outcome | n_SNPs | Model | Beta | 95% CI | *P* value |
| --- | --- | --- | --- | --- | --- | --- |
| IBD | ER | 55 | IVW | -0.036 | -0.087, 0.015 | 0.161 |
|  |  |  | MR Egger | 0.041 | -0.099, 0.181 | 0.568 |
|  |  |  | Weighted median | -0.025 | -0.102, 0.053 | 0.529 |
| CD |  | 45 | IVW | -0.041 | -0.081, -0.001 | **0.046*** |
|  |  |  | MR Egger | -0.040 | -0.133, 0.053 | 0.402 |
|  |  |  | Weighted median | -0.029 | -0.089, 0.032 | 0.350 |
| UC |  | 32 | IVW | 0.022 | -0.029, 0.073 | 0.391 |
|  |  |  | MR Egger | 0.037 | -0.102, 0.176 | 0.608 |
|  |  |  | Weighted median | 0.042 | -0.033, 0.117 | 0.277 |
| IBD | PRLR | 63 | IVW | -0.029 | -0.074, 0.015 | 0.192 |
|  |  |  | MR Egger | -0.053 | -0.165, 0.059 | 0.356 |
|  |  |  | Weighted median | -0.033 | -0.101, 0.035 | 0.342 |
| CD |  | 52 | IVW | -0.028 | -0.066, 0.009 | 0.140 |
|  |  |  | MR Egger | -0.029 | -0.115, 0.057 | 0.510 |
|  |  |  | Weighted median | -0.016 | -0.074, 0.043 | 0.600 |
| UC |  | 37 | IVW | -0.030 | -0.076, 0.017 | 0.208 |
|  |  |  | MR Egger | -0.046 | -0.159, 0.066 | 0.424 |
|  |  |  | Weighted median | -0.030 | -0.101, 0.040 | 0.402 |
| IBD | SHBG | 63 | IVW | 0.002 | -0.003, 0.006 | 0.455 |
|  |  |  | MR Egger | 0.004 | -0.007, 0.015 | 0.454 |
|  |  |  | Weighted median | 0.001 | -0.003, 0.004 | 0.700 |
| CD |  | 52 | IVW | 0.000 | -0.003, 0.004 | 0.808 |
|  |  |  | MR Egger | -0.002 | -0.011, 0.007 | 0.709 |
|  |  |  | Weighted median | 0.001 | -0.002, 0.003 | 0.702 |
| UC |  | 37 | IVW | 0.002 | -0.003, 0.007 | 0.517 |
|  |  |  | MR Egger | 0.008 | -0.004, 0.020 | 0.184 |
|  |  |  | Weighted median | 0.001 | -0.003, 0.004 | 0.713 |

**P*<0.05. Abbreviations: IBD, inflammatory bowel disease; CD, Crohn’s disease; UC, ulcerative colitis; ER, estrogen receptor; PRLR, prolactin receptor; SHBG, sex hormone-binding globulin; CI, confidence interval; IVW, inverse variance weighted.

**Table S16. Heterogeneity and pleiotropy tests for the associations between IBD/CD/UC and ER/PRLR/SHBG.**

| Exposure | Outcome | Cochran’s Q test | | MR PRESSO test | | MR Egger test | |
| --- | --- | --- | --- | --- | --- | --- | --- |
|  |  | Q | *P* value | Global test  *P* value | Distortion test  *P* value | Intercept | *P* value |
| IBD | ER | 62.062 | 0.211 | 0.227 | - | -0.013 | 0.250 |
| CD |  | 44.801 | 0.438 | 0.458 | - | -0.000 | 0.988 |
| UC |  | 27.804 | 0.631 | 0.638 | - | -0.003 | 0.828 |
| IBD | PRLR | 51.379 | 0.830 | 0.833 | - | 0.004 | 0.652 |
| CD |  | 34.712 | 0.961 | 0.966 | - | 0.000 | 0.986 |
| UC |  | 35.262 | 0.504 | 0.532 | - | 0.004 | 0.753 |
| IBD | SHBG | 408.311 | <0.001 | <0.001 | 0.043 | -0.000 | 0.618 |
| CD |  | 383.414 | <0.001 | <0.001 | 0.168 | 0.001 | 0.594 |
| UC |  | 282.854 | <0.001 | <0.001 | 0.491 | -0.001 | 0.241 |

Abbreviations: IBD, inflammatory bowel disease; CD, Crohn’s disease; UC, ulcerative colitis; ER, estrogen receptor; PRLR, prolactin receptor; SHBG, sex hormone-binding globulin.

**Table S17. Sex-stratified causal effects of IBD/CD/UC on E2 using different models.**

| Exposure | Outcome | Out_sex | n_SNPs | Model | Beta | 95% CI | *P* value |
| --- | --- | --- | --- | --- | --- | --- | --- |
| IBD | E2 | Male | 58 | IVW | 0.003 | -0.027, 0.033 | 0.841 |
|  |  |  |  | MR Egger | 0.012 | -0.061, 0.085 | 0.743 |
|  |  |  |  | Weighted median | 0.019 | -0.017, 0.055 | 0.306 |
|  |  | Female | 58 | IVW | -0.007 | -0.033, 0.019 | 0.581 |
|  |  |  |  | MR Egger | 0.043 | -0.019, 0.105 | 0.178 |
|  |  |  |  | Weighted median | 0.002 | -0.033, 0.037 | 0.912 |
| CD |  | Male | 42 | IVW | -0.007 | -0.034, 0.020 | 0.624 |
|  |  |  |  | MR Egger | -0.015 | -0.073, 0.042 | 0.603 |
|  |  |  |  | Weighted median | -0.002 | -0.035, 0.032 | 0.923 |
|  |  | Female | 42 | IVW | -0.015 | -0.039, 0.008 | 0.198 |
|  |  |  |  | MR Egger | 0.008 | -0.042, 0.058 | 0.767 |
|  |  |  |  | Weighted median | -0.003 | -0.036, 0.031 | 0.868 |
| UC |  | Male | 31 | IVW | 0.006 | -0.027, 0.040 | 0.705 |
|  |  |  |  | MR Egger | 0.006 | -0.071, 0.083 | 0.885 |
|  |  |  |  | Weighted median | 0.020 | -0.019, 0.060 | 0.311 |
|  |  | Female | 31 | IVW | 0.001 | -0.023, 0.025 | 0.927 |
|  |  |  |  | MR Egger | 0.034 | -0.021, 0.088 | 0.231 |
|  |  |  |  | Weighted median | 0.009 | -0.025, 0.043 | 0.589 |

Abbreviations: IBD, inflammatory bowel disease; CD, Crohn’s disease; UC, ulcerative colitis; E2, estradiol; CI, confidence interval; IVW, inverse variance weighted.

**Table S18. Sex-stratified causal effects of IBD/CD/UC on PROG using different models.**

| Exposure | Outcome | Out_sex | n_SNPs | Model | Beta | Beta 95% CI | *P* value |
| --- | --- | --- | --- | --- | --- | --- | --- |
| IBD | PROG | Male | 55 | IVW | 0.017 | -0.013, 0.046 | 0.275 |
|  |  |  |  | MR Egger | 0.010 | -0.073, 0.092 | 0.819 |
|  |  |  |  | Weighted median | 0.011 | -0.033, 0.056 | 0.616 |
|  |  | Female | 49 | IVW | -0.090 | -0.162, -0.017 | **0.015*** |
|  |  |  |  | MR Egger | -0.049 | -0.244, 0.146 | 0.625 |
|  |  |  |  | Weighted median | -0.132 | -0.242, -0.021 | **0.019*** |
| CD |  | Male | 47 | IVW | 0.016 | -0.008, 0.041 | 0.188 |
|  |  |  |  | MR Egger | 0.024 | -0.032, 0.080 | 0.400 |
|  |  |  |  | Weighted median | 0.011 | -0.029, 0.050 | 0.594 |
|  |  | Female | 40 | IVW | -0.038 | -0.106, 0.030 | 0.271 |
|  |  |  |  | MR Egger | -0.034 | -0.186, 0.117 | 0.659 |
|  |  |  |  | Weighted median | -0.095 | -0.186, -0.004 | **0.040*** |
| UC |  | Male | 30 | IVW | -0.001 | -0.037, 0.035 | 0.948 |
|  |  |  |  | MR Egger | 0.044 | -0.055, 0.142 | 0.392 |
|  |  |  |  | Weighted median | 0.015 | -0.034, 0.063 | 0.555 |
|  |  | Female | 26 | IVW | -0.100 | -0.180, -0.019 | **0.015*** |
|  |  |  |  | MR Egger | -0.123 | -0.334, 0.088 | 0.263 |
|  |  |  |  | Weighted median | -0.063 | -0.178, 0.052 | 0.283 |

**P*<0.05. Abbreviations: IBD, inflammatory bowel disease; CD, Crohn’s disease; UC, ulcerative colitis; PROG, progesterone; CI, confidence interval; IVW, inverse variance weighted.

**Table S19. Sex-stratified causal effects of IBD/CD/UC on BAT using different models.**

| Exposure | Outcome | Out_sex | n_SNPs | Model | Beta | 95% CI | *P* value |
| --- | --- | --- | --- | --- | --- | --- | --- |
| IBD | BAT | Male | 63 | IVW | -0.007 | -0.017, 0.003 | 0.195 |
|  |  |  |  | MR Egger | 0.014 | -0.011, 0.039 | 0.284 |
|  |  |  |  | Weighted median | 0.005 | -0.004, 0.014 | 0.301 |
|  |  | Female | 63 | IVW | 0.001 | -0.007, 0.009 | 0.831 |
|  |  |  |  | MR Egger | 0.001 | -0.019, 0.022 | 0.894 |
|  |  |  |  | Weighted median | 0.003 | -0.005, 0.011 | 0.490 |
| CD |  | Male | 52 | IVW | -0.011 | -0.021, -0.001 | **0.036*** |
|  |  |  |  | MR Egger | 0.010 | -0.013, 0.032 | 0.401 |
|  |  |  |  | Weighted median | -0.000 | -0.009, 0.008 | 0.918 |
|  |  | Female | 52 | IVW | 0.001 | -0.007, 0.009 | 0.792 |
|  |  |  |  | MR Egger | 0.003 | -0.016, 0.022 | 0.775 |
|  |  |  |  | Weighted median | 0.002 | -0.005, 0.008 | 0.625 |
| UC |  | Male | 37 | IVW | -0.004 | -0.013, 0.006 | 0.450 |
|  |  |  |  | MR Egger | -0.001 | -0.024, 0.021 | 0.906 |
|  |  |  |  | Weighted median | -0.000 | -0.010, 0.010 | 0.999 |
|  |  | Female | 37 | IVW | 0.000 | -0.007, 0.007 | 0.989 |
|  |  |  |  | MR Egger | -0.001 | -0.019, 0.017 | 0.918 |
|  |  |  |  | Weighted median | 0.000 | -0.008, 0.008 | 0.986 |

**P*<0.05. Abbreviations: IBD, inflammatory bowel disease; CD, Crohn’s disease; UC, ulcerative colitis; BAT, bioavailable testosterone; CI, confidence interval; IVW, inverse variance weighted.

**Table S20. Sex-stratified causal effects of IBD/CD/UC on TT using different models.**

| Exposure | Outcome | Out_sex | n_SNPs | Model | Beta | 95% CI | *P* value |
| --- | --- | --- | --- | --- | --- | --- | --- |
| IBD | TT | Male | 63 | IVW | 0.002 | -0.009, 0.012 | 0.769 |
|  |  |  |  | MR Egger | 0.023 | -0.003, 0.049 | 0.088 |
|  |  |  |  | Weighted median | 0.011 | 0.002, 0.020 | **0.020*** |
|  |  | Female | 63 | IVW | -0.002 | -0.011, 0.007 | 0.632 |
|  |  |  |  | MR Egger | -0.004 | -0.027, 0.019 | 0.736 |
|  |  |  |  | Weighted median | -0.003 | -0.012, 0.006 | 0.471 |
| CD |  | Male | 52 | IVW | -0.006 | -0.016, 0.005 | 0.312 |
|  |  |  |  | MR Egger | 0.008 | -0.016, 0.033 | 0.516 |
|  |  |  |  | Weighted median | 0.005 | -0.003, 0.013 | 0.228 |
|  |  | Female | 52 | IVW | -0.001 | -0.012, 0.009 | 0.793 |
|  |  |  |  | MR Egger | -0.009 | -0.033, 0.016 | 0.490 |
|  |  |  |  | Weighted median | -0.001 | -0.008, 0.006 | 0.762 |
| UC |  | Male | 37 | IVW | 0.005 | -0.003, 0.014 | 0.215 |
|  |  |  |  | MR Egger | 0.019 | -0.002, 0.039 | 0.083 |
|  |  |  |  | Weighted median | 0.011 | 0.002, 0.020 | **0.017*** |
|  |  | Female | 37 | IVW | -0.002 | -0.009, 0.005 | 0.552 |
|  |  |  |  | MR Egger | 0.001 | -0.016, 0.019 | 0.881 |
|  |  |  |  | Weighted median | -0.002 | -0.010, 0.007 | 0.697 |

**P*<0.05. Abbreviations: IBD, inflammatory bowel disease; CD, Crohn’s disease; UC, ulcerative colitis; TT, total testosterone; CI, confidence interval; IVW, inverse variance weighted.

**Table S21. Sex-stratified causal effects of IBD/CD/UC on SHBG using different models.**

| Exposure | Outcome | Out_sex | n_SNPs | Model | Beta | 95% CI | *P* value |
| --- | --- | --- | --- | --- | --- | --- | --- |
| IBD | SHBG | Male | 63 | IVW | 0.004 | -0.000, 0.008 | 0.053 |
|  |  |  |  | MR Egger | 0.005 | -0.006, 0.015 | 0.388 |
|  |  |  |  | Weighted median | 0.001 | -0.003, 0.005 | 0.644 |
|  |  | Female | 63 | IVW | -0.000 | -0.005, 0.005 | 0.958 |
|  |  |  |  | MR Egger | 0.002 | -0.011, 0.015 | 0.762 |
|  |  |  |  | Weighted median | -0.000 | -0.005, 0.004 | 0.925 |
| CD |  | Male | 52 | IVW | 0.002 | -0.002, 0.005 | 0.377 |
|  |  |  |  | MR Egger | -0.001 | -0.010, 0.007 | 0.782 |
|  |  |  |  | Weighted median | 0.001 | -0.003, 0.004 | 0.753 |
|  |  | Female | 52 | IVW | -0.000 | -0.005, 0.004 | 0.902 |
|  |  |  |  | MR Egger | -0.003 | -0.014, 0.008 | 0.601 |
|  |  |  |  | Weighted median | 0.000 | -0.004, 0.004 | 0.954 |
| UC |  | Male | 37 | IVW | 0.005 | 0.000, 0.009 | **0.046*** |
|  |  |  |  | MR Egger | 0.008 | -0.003, 0.019 | 0.185 |
|  |  |  |  | Weighted median | 0.003 | -0.001, 0.007 | 0.176 |
|  |  | Female | 37 | IVW | -0.001 | -0.007, 0.005 | 0.781 |
|  |  |  |  | MR Egger | 0.007 | -0.007, 0.021 | 0.358 |
|  |  |  |  | Weighted median | -0.000 | -0.005, 0.005 | 0.949 |

**P*<0.05. Abbreviations: IBD, inflammatory bowel disease; CD, Crohn’s disease; UC, ulcerative colitis; SHBG, sex hormone-binding globulin; CI, confidence interval; IVW, inverse variance weighted.

**Table S22. Sex-stratified causal effects of IBD/CD/UC on AMH using different models.**

| Exposure | Outcome | Out_sex | n_SNPs | Model | Beta | 95% CI | *P* value |
| --- | --- | --- | --- | --- | --- | --- | --- |
| IBD | AMH | Female | 57 | IVW | 0.000 | -0.027, 0.028 | 0.977 |
|  |  |  |  | MR Egger | -0.012 | -0.089, 0.064 | 0.756 |
|  |  |  |  | Weighted median | 0.002 | -0.041, 0.045 | 0.931 |
| CD |  | Female | 48 | IVW | 0.012 | -0.012, 0.036 | 0.323 |
|  |  |  |  | MR Egger | -0.009 | -0.064, 0.045 | 0.738 |
|  |  |  |  | Weighted median | 0.003 | -0.031, 0.037 | 0.866 |
| UC |  | Female | 34 | IVW | -0.011 | -0.039, 0.017 | 0.451 |
|  |  |  |  | MR Egger | 0.052 | -0.035, 0.139 | 0.250 |
|  |  |  |  | Weighted median | 0.002 | -0.040, 0.044 | 0.917 |

**P*<0.05. Abbreviations: IBD, inflammatory bowel disease; CD, Crohn’s disease; UC, ulcerative colitis; AMH, anti-Müllerian hormone; CI, confidence interval; IVW, inverse variance weighted.

**Table S23. Heterogeneity and pleiotropy tests for the sex-stratified associations between IBD/CD/UC and E2/PROG/BAT/TT/SHBG/AMH.**

| Exposure | Outcome | Out_sex | Cochran’s Q test | | MR PRESSO test | | MR Egger test | |
| --- | --- | --- | --- | --- | --- | --- | --- | --- |
|  |  |  | Q | *P* value | Global test  *P* value | Distortion test  *P* value | Intercept | *P* value |
| IBD | E2 | Male | 89.095 | 0.004 | 0.005 | 0.040 | -0.002 | 0.787 |
|  |  | Female | 77.375 | 0.038 | 0.038 | 0.057 | -0.009 | 0.086 |
| CD |  | Male | 66.290 | 0.007 | 0.007 | - | 0.002 | 0.740 |
|  |  | Female | 57.167 | 0.048 | 0.053 | - | -0.006 | 0.312 |
| UC |  | Male | 50.423 | 0.011 | 0.010 | - | 0.000 | 0.985 |
|  |  | Female | 25.704 | 0.690 | 0.701 | - | -0.007 | 0.199 |
| IBD | PROG | Male | 44.461 | 0.819 | 0.839 | - | 0.001 | 0.863 |
|  |  | Female | 50.521 | 0.374 | 0.367 | - | -0.007 | 0.661 |
| CD |  | Male | 37.425 | 0.812 | 0.810 | - | -0.002 | 0.763 |
|  |  | Female | 54.797 | 0.048 | 0.051 | - | -0.001 | 0.958 |
| UC |  | Male | 35.465 | 0.190 | 0.184 | - | -0.009 | 0.346 |
|  |  | Female | 19.687 | 0.763 | 0.777 | - | 0.005 | 0.817 |
| IBD | BAT | Male | 214.889 | <0.001 | <0.001 | 0.531 | -0.004 | 0.086 |
|  |  | Female | 201.377 | <0.001 | <0.001 | 0.814 | -0.000 | 0.957 |
| CD |  | Male | 240.335 | <0.001 | <0.001 | 0.955 | -0.005 | 0.051 |
|  |  | Female | 229.880 | <0.001 | <0.001 | 0.245 | -0.000 | 0.849 |
| UC |  | Male | 93.734 | <0.001 | <0.001 | 0.571 | -0.000 | 0.834 |
|  |  | Female | 83.599 | <0.001 | <0.001 | - | 0.000 | 0.905 |
| IBD | TT | Male | 232.438 | <0.001 | <0.001 | 0.778 | -0.004 | 0.084 |
|  |  | Female | 211.225 | <0.001 | <0.001 | 0.631 | 0.000 | 0.870 |
| CD |  | Male | 279.477 | <0.001 | <0.001 | 0.020 | -0.003 | 0.226 |
|  |  | Female | 311.905 | <0.001 | <0.001 | 0.738 | 0.002 | 0.521 |
| UC |  | Male | 83.914 | <0.001 | <0.001 | 0.681 | -0.003 | 0.175 |
|  |  | Female | 65.873 | 0.002 | 0.001 | 0.085 | -0.001 | 0.665 |
| IBD | SHBG | Male | 226.443 | <0.001 | <0.001 | 0.537 | -0.001 | 0.909 |
|  |  | Female | 255.532 | <0.001 | <0.001 | 0.599 | -0.000 | 0.725 |
| CD |  | Male | 207.079 | <0.001 | <0.001 | 0.983 | 0.001 | 0.462 |
|  |  | Female | 241.034 | <0.001 | <0.001 | 0.869 | 0.001 | 0.601 |
| UC |  | Male | 139.238 | <0.001 | <0.001 | 0.447 | -0.001 | 0.565 |
|  |  | Female | 172.349 | <0.001 | <0.001 | 0.715 | -0.002 | 0.257 |
| IBD | AMH | Female | 63.788 | 0.222 | 0.221 | - | 0.002 | 0.730 |
| CD |  | Female | 57.867 | 0.133 | 0.142 | - | 0.005 | 0.397 |
| UC |  | Female | 26.542 | 0.779 | 0.782 | - | -0.012 | 0.144 |

Abbreviations: IBD, inflammatory bowel disease; CD, Crohn’s disease; UC, ulcerative colitis; E2, estradiol; PROG, progesterone; BAT, bioavailable testosterone; TT, total testosterone; SHBG, sex hormone-binding globulin; AMH, anti-Müllerian hormone.
